# Supplementary material for: Trends in the prevalence and treatment of depressive symptoms in Peru: a population-based study
Source: BMJ Open. 2020 Jul 19;10(7):e036777. doi: 10.1136/bmjopen-2020-036777 (PMC7371215; doi:10.1136/bmjopen-2020-036777)
Supplement: Supplementary data [file bmjopen-2020-036777supp001.pdf]

Supplement 1. Descriptive characteristics of the participants not included in the study (2014-2018).

		2014		2015		2016		2017		2018	
		n	%	*n*	%	*n*	%	*n*	%	*n*	%
Sex	Male	0	0.0%	1	0.1%	0	0.0%	0	0.0%	0	0.00%
	Female	0	0.0%	8	0.7%	0	0.0%	1	0.1%	0	0.00%
	Missing	1,160	100.0%	1,101	99.2%	1,166	100.0%	880	99.9%	912	100.00%
Age	15-34	0	0.0%	4	0.3%	0	0.0%	0	0.0%	0	0.00%
	35-54	0	0.0%	3	0.3%	0	0.0%	0	0.0%	0	0.00%
	55-74	0	0.0%	1	0.1%	0	0.0%	0	0.0%	0	0.00%
	75+	0	0.0%	1	0.1%	0	0.0%	74	0.1%	0	0.00%
	Missing	1,160	100.0%	1,101	99.2%	1,166	100.0%	880	99.9%	912	100.00%
Area	Rural	261	22.5%	289	26.0%	253	21.7%	194	22.0%	175	19.2%
	Urban	899	77.5%	821	74.0%	913	78.3%	687	78.0%	737	80.8%
	Missing	0	0.0%	0	0.0%	0	0.0%	0	0.0%	0	0.00%
Wealth index	Very low	207	17.8%	261	23.5%	214	18.4%	181	20.5%	155	17.0%
	Low	205	17.7%	201	18.1%	209	17.9%	152	17.3%	162	17.8%
	Middle	230	19.8%	192	17.3%	232	19.9%	152	17.3%	199	21.8%
	High	241	20.8%	225	20.3%	258	22.1%	204	23.2%	159	17.4%
	Very high	277	23.9%	231	20.8%	253	21.7%	192	21.8%	237	26.0%
	Missing	0	0.0%	0	0.0%	0	0.0%	0	0.0%	0	0.00%
