# Supplementary material for: Trends in the prevalence and treatment of depressive symptoms in Peru: a population-based study
Source: BMJ Open. 2020 Jul 19;10(7):e036777. doi: 10.1136/bmjopen-2020-036777 (PMC7371215; doi:10.1136/bmjopen-2020-036777)
Supplement: Supplementary data [file bmjopen-2020-036777supp002.pdf]

Supplement 2. Factor loads, goodness-of-fit indices, and internal consistency coefficients of the modified version of the PHQ-9 to evaluate depressive symptoms in the past year.

	2014	2015	2016	2017	2018
1. Anhedonia	0.63	0.66	0.69	0.69	0.69
2. Depressed mood	0.66	0.67	0.71	0.71	0.68
3. Sleep disturbance	0.62	0.65	0.66	0.66	0.64
4. Fatigue	0.64	0.68	0.68	0.68	0.70
5. Appetite changes	0.62	0.64	0.63	0.63	0.63
6. Feelings of worthlessness	0.66	0.67	0.68	0.68	0.71
7. Concentration difficulties	0.65	0.67	0.67	0.67	0.68
8. Psychomotor disturbances	0.55	0.59	0.60	0.60	0.58
9. Thoughts of death	0.51	0.56	0.55	0.55	0.54
χ^2	660.6	768.0	864.0	864.2	679.2
CFI	0.918	0.925	0.912	0.920	0.921
TLI	0.900	0.901	0.898	0.901	0.901
RMSEA [CI 90%]	0.051 [0.049 - 0.053]	0.053 [0.051 - 0.055]	0.058 [0.056 - 0.060]	0.059 [0.058 - 0.061]	0.050 [0.051 - 0.053]
α	0.85	0.86	0.87	0.87	0.87
ω	0.85	0.87	0.87	0.87	0.86
r with the PHQ-9 (two-week)	0.60	0.61	0.61	0.61	0.60
