# Supplementary material for: Trends in the prevalence and treatment of depressive symptoms in Peru: a population-based study
Source: BMJ Open. 2020 Jul 19;10(7):e036777. doi: 10.1136/bmjopen-2020-036777 (PMC7371215; doi:10.1136/bmjopen-2020-036777)
Supplement: Supplementary data [file bmjopen-2020-036777supp003.pdf]

Supplement 3. Age-standardised prevalence of depressive symptoms and proportion of treatment in Peru by region in 2018 (with cut-off point 15).

Region	Age-standardised prevalence of Depressive symptoms in the last two week *	Age-standardised prevalence of Depressive symptoms in the last year *	Proportion of People with depressive symptoms receiving treatment **
Amazonas	2.19%	5.63%	11.77%
Ancash	4.03%	9.95%	10.67%
Apurímac	2.52%	7.81%	16.63%
Arequipa	1.20%	4.19%	19.51%
Ayacucho	3.68%	8.93%	8.27%
Cajamarca	2.47%	5.54%	11.48%
Callao	1.70%	4.51%	38.51%
Cusco	4.36%	12.36%	9.74%
Huancavelica	6.10%	18.47%	5.89%
Huánuco	3.62%	7.95%	17.87%
Ica	2.99%	5.78%	15.42%
Junín	3.12%	6.87%	9.79%
La Libertad	1.41%	4.15%	28.52%
Lambayeque	0.40%	2.10%	17.19%
Lima	1.69%	5.40%	15.77%
Loreto	0.80%	2.20%	2.38%
Madre de Dios	2.12%	3.63%	22.03%
Moquegua	0.84%	3.36%	10.07%
Pasco	1.27%	4.61%	24.52%
Piura	3.21%	7.91%	14.08%
Puno	6.39%	13.71%	7.12%
San Martín	2.05%	5.16%	12.92%
Tacna	3.86%	7.21%	12.34%
Tumbes	0.88%	2.52%	13.03%
Ucayali	0.75%	3.39%	14.51%

Note: Two-stage sample design was taken into account for percentage estimations. * The analysis considered the total of Peruvian population. ** An analysis is done by subgroups, considering only people who have depressive symptoms.
