# Supplementary material for: Trends in the prevalence and treatment of depressive symptoms in Peru: a population-based study
Source: BMJ Open. 2020 Jul 19;10(7):e036777. doi: 10.1136/bmjopen-2020-036777 (PMC7371215; doi:10.1136/bmjopen-2020-036777)
Supplement: Author's manuscript [file bmjopen-2020-036777.draft_revisions.pdf]

BMJ Open is committed to open peer review. As part of this commitment we make the peer review history of every article we publish publicly available.

When an article is published we post the peer reviewers' comments and the authors' responses online. We also post the versions of the paper that were used during peer review. These are the versions that the peer review comments apply to.

The versions of the paper that follow are the versions that were submitted during the peer review process. They are not the versions of record or the final published versions. They should not be cited or distributed as the published version of this manuscript.

BMJ Open is an open access journal and the full, final, typeset and author-corrected version of record of the manuscript is available on our site with no access controls, subscription charges or pay-per-view fees (http://bmjopen.bmj.com).

If you have any questions on BMJ Open's open peer review process please email info.bmjopen@bmj.com

Trends in the prevalence and treatment of depressive symptoms in Peru

Journal:	*BMJ Open*
Manuscript ID	bmjopen-2020-036777
Article Type:	Original research
Date Submitted by the Author:	06-Jan-2020

Complete List of Authors:	Villarreal-Zegarra, David; Instituto Peruano de Orientación Psicológica, ; Universidad Peruana Cayetano Heredia, CRONICAS Center of Excellence in Chronic Diseases<br>Cabrera-Alva, Milagros; Instituto Peruano de Orientación Psicológica<br>Carrillo-Larco, Rodrigo; Universidad Peruana Cayetano Heredia, CRONICAS Center of Excellence in Chronic Diseases; Imperial College London School of Public Health, Department of Epidemiology and Biostatistics<br>Bernabe-Ortiz, Antonio; Universidad Peruana Cayetano Heredia, CRONICAS Centre of Excellence in Chronic Diseases; Universidad Científica del Sur

Keywords:	Depression & mood disorders < PSYCHIATRY, EPIDEMIOLOGY, PUBLIC HEALTH

SCHOLARONE™
Manuscripts

I, the Submitting Author has the right to grant and does grant on behalf of all authors of the Work (as defined in the below author licence), an exclusive licence and/or a non-exclusive licence for contributions from authors who are: i) UK Crown employees; ii) where BMJ has agreed a CC-BY licence shall apply, and/or iii) in accordance with the terms applicable for US Federal Government officers or employees acting as part of their official duties; on a worldwide, perpetual, irrevocable, royalty-free basis to BMJ Publishing Group Ltd ("BMJ") its licensees and where the relevant Journal is co-owned by BMJ to the co-owners of the Journal, to publish the Work in this journal and any other BMJ products and to exploit all rights, as set out in our licence.

The Submitting Author accepts and understands that any supply made under these terms is made by BMJ to the Submitting Author unless you are acting as an employee on behalf of your employer or a postgraduate student of an affiliated institution which is paying any applicable article publishing charge ("APC") for Open Access articles. Where the Submitting Author wishes to make the Work available on an Open Access basis (and intends to pay the relevant APC), the terms of reuse of such Open Access shall be governed by a Creative Commons licence – details of these licences and which Creative Commons licence will apply to this Work are set out in our licence referred to above.

Other than as permitted in any relevant BMJ Author's Self Archiving Policies, I confirm this Work has not been accepted for publication elsewhere, is not being considered for publication elsewhere and does not duplicate material already published. I confirm all authors consent to publication of this Work and authorise the granting of this licence.

For peer review only

Trends in the prevalence and treatment of depressive symptoms in Peru

Authors

David Villarreal-Zegarra [1, 2]

Milagros Cabrera-Alva [2]

Rodrigo Carrillo-Larco [1, 3, 4]

Antonio Bernabe-Ortiz [1, 5]

Affiliation

[1] CRONICAS Center of Excellence in Chronic Diseases, Universidad Peruana Cayetano Heredia, Lima, Peru.

[2] Instituto Peruano de Orientación Psicológica, Lima, Peru.

[3] Department of Epidemiology and Biostatistics, School of Public Health, Imperial College London, London, United Kingdom.

[4] Centro de Estudios de Población, Universidad Católica los Ángeles de Chimbote (ULADECH), Chimbote, Peru.

[5] Universidad Científica del Sur, Lima, Peru.

Corresponding author

David Villarreal-Zegarra

E-mail: davidvillarreal@ipops.pe

Avenida Armendáriz 445, Miraflores, Lima 18, Perú

Telf: (+511) 241-6978

Number of Figures: 2

Number of Tables: 2

Number of Supplements: 3

Number of words: 3826

For peer review only

ARTICLE SUMMARY

Objectives: This study aimed to estimate the trends in the prevalence and treatment of depressive symptoms using nationally representative surveys in Peru from 2014 to 2018.

Design: A cross-sectional study was conducted using secondary data (five-years) of the Demographic and Health Survey of Peru (ENDES Spanish acronym), which is carried out annually and is representative at the country level.

Setting: The study was conducted in Peru.

Participants: The participants in each year of measurement were different and the sampling used was probabilistic in two stages. Men and women, aged 15 years or more, were included.

Main outcome measures: Two versions of the Patient's Health Questionnaire (PHQ-9) that focused on the presence of depressive symptoms in the last two weeks and in the last year were used. The age-standardized prevalence was estimated using the World Health Organization population as the reference population.

Results: A total of 161,061 participants were included. There was no evidence of a trend across the observation period. In 2018, the age-standardized prevalence in the last two weeks and in the last year was 2.3% (95%CI: 2.1%-2.5%) and 6.2% (95%CI: 5.8%-6.6%), respectively. People with depressive symptoms in the last year who reported receiving treatment was 14.4% (95%CI: 12.2%-16.9%). The percentage of treatment was lower in people with a low socioeconomic level.

Conclusions: Although there was no evidence of a decreasing or increasing trend in the last five years; one in ten people exhibited depressive symptoms during the last year and one in twenty in the last two weeks. With evidence that roughly one in ten people with depressive symptoms received treatment, efforts should be deployed to meet the diagnosis and treatment gap in the population.

Keyword: Depression; Epidemiology; Mood disorders; Treatment; Primary care.

For peer review only

STRENGTHS AND LIMITATIONS OF THIS STUDY

- ✓ The strength of the study is the analysis of five years of information from nationally-representative surveys of the Peruvian population.
- ✓ The period evaluated was short (five years). If prevalence or treatment were to increase, this increase would most likely be small but sustained annually, so it would be necessary to have a greater number of measurements (10 or 20 years) in order to be able to identify a trend of higher or a decrease of depression symptoms.
- ✓ Although the evaluation of depressive symptoms was performed using a valid tool, this does not replace an evaluation conducted by a psychiatrist; thus, misclassification may be an issue. However, any misclassification present in the study may be non-differential because the same methods were used to evaluate each year and in each group.
- ✓ Another element that could generate a misclassification is the cut-off point used to classify people with depressive symptoms (PHQ-9's scores ≥15). There is evidence suggesting the use of the cut-off point of 15 in PHQ-9 since the severity of depressive symptoms is consistent with a greater depression that normally requires medication.

For peer review only

HIGHLIGHTS

- ✓ In Peru, between 2014 and 2018, no changes were observed in the age-standardized prevalence of depressive symptoms (two weeks neither a year prior to evaluation moment) neither in the percentage of people who reported having received treatment to improve these symptoms.
- ✓ In 2018, the age-standardized prevalence in the last two weeks and in the last year before the evaluation moment was 2.3% and 6.2%, respectively. Among the individuals reporting depressive symptoms in the last year, 14.4% reported receiving treatment for depressive symptoms.
- ✓ The percentage of treatment of depressive symptoms in the last year is much lower among people of a very low socioeconomic status (6.2% in 2018) in comparison to those of a very high socioeconomic status (26.1% in 2018).

For peer review only

INTRODUCTION

Depression is a significant public health issue that affects approximately 322 million people worldwide [1], and has high social and economic cost implications [2 3]. Based on projections, it is estimated that depression will be the leading cause of years lost to disability (DALY´s) by 2030 [4]. Epidemiological surveillance of the trends in depressive symptoms is of great relevance to public health as changes in the prevalence and treatment can aid researchers and stakeholders make more informed decisions in how to address this challenge.

The prevalence of depressive symptoms can vary depending on several biological, sociodemographic or lifestyle factors. For example, women [5], older adults [6], and people with a low socioeconomic status [7] have a higher prevalence of depressive symptoms. In addition to this, treatment of depressive symptoms is also associated with health system accessibility. A systematic review identified that lack of human resources, centralization of the health system, and integration in primary care, are barriers to receiving appropriate treatment of depressive symptoms [8].

Currently, there is no consensus on the trends of the prevalence of depressive symptoms. A meta-analysis of 116 epidemiological investigations during the years 1990 and 2010 did not reveal changes in the prevalence of major depressive disorder [9]. However, the Global Burden of Disease Studies assessing trends in previous years [10 11] and other longitudinal studies showed an increase in the prevalence of major depressive disorder [12 13]. These heterogeneous results can be attributed to inter-country variations of several demographic and socioeconomic factors as well as the different criteria used to define depression in research studies. Due to the lack of global consensus, country-specific estimates and trends are important and can inform local policies and guidelines.

In Peru, the prevalence of depressive symptoms ranges from 14% in urban areas to 12.5% in rural areas [14]; whereas the prevalence of major depressive disorders reported in the last year was 2.7%; of which a third receives minimally adequate treatment [15]. Moreover, access to treatment for those suffering from depressive disorders is very limited in low and middle-income countries; with only 1 in every 27 patients with major depressive disorder managing to receive treatment [15]. Since 2015, Peru has been implementing mental health reforms that focus on primary care and the redistribution of its resources from hospital care to community care centers [16].

It is therefore fundamental to have up-to-date metrics on the prevalence and level of treament coverage of depressive symptoms in order to determine an appropriate response to this disease. This study aims to estimate trends in prevalence and treatment of depressive symptoms using nationally representative surveys in Peru between 2014 and 2018.

METHODS

Study design

Data from the National Demographic and Health Survey of Peru (ENDES – Spanish acronym), which collects information on a number of variables including poverty, fertility, violence, and health, was utilized in this study. The first ENDES was conducted in 1996, the second in 2000, and the third in 2004, before being carried out annually from that year on after. Since 2013, ENDES began to incorporate questions regarding mental health into its evaluation; however it initially only used a very small sub-sample that was not representative of the general population. From 2014 onwards, the data collected on mental health was nationally representative. Our study therefore decided to use data collected by ENDES over this five year period (between the years 2014 and 2018).

It is additionally important to note that the participants from whom the data was being collected from in the annual ENDES evaluations were different each year, and therefore the data being evaluated in this study came from five different groups (one from each year of evaluation).

For peer review only

Participants

The sampling used was probabilistic in two stages and representative at the country level. In rural areas, the primary sampling units were comprised of groups ranging from 500 to 2,000 people, whereas the secondary units included households. Contrastingly, the primary sampling units in urban areas were blocks or groups of blocks of more than 2,000 individuals with an average of 140 households. Secondary sampling units were the same as in rural settings [17]. Details on the sampling process can be found in the technical documents of the ENDES [18].

Participants of both sexes, who were of more than 15 years of age, living in both rural and urban areas and from every region in Peru, were included in ENDES. This study only included participants with complete data on the PHQ-9 and the sociodemographic variables of interest (sex, age, area, economic level, region, and year of evaluation).

Variables

Depressive symptoms were evaluated using two different approaches (PHQ-9 and a modified-version of PHQ-9) which looked at participants' responses to experiencing symptoms of depression in the last two weeks and in the last year before the evaluation moment. The first approach enabled the identification of the number of cases with depressive symptoms two weeks prior to the last ENDES evaluation whilst the second aided in the identification of the occurrence of cases showing symptoms of depression over a longer period of time. In addition to this, the treatment of depressive symptoms and other socio-demographic variables were evaluated based on self-reported questionnaires.

Depressive symptomatology in the last two weeks

Depressive symptomatology was evaluated using the Patient's Health Questionnaire (PHQ-9), available in the ENDES 2014-2018. The PHQ-9 was adapted for the population in Peru and had shown optimal values of reliability and validity when tested [19]. This tool has nine items based on the DSM-IV criteria and qualifies the response options from zero to three. PHQ-9 can therefore present a minimum score of 0 and a maximum score of 27. Depending on the total score, the PHQ-9 results may be: without indicators of depressive symptoms (score 0 to 4); mild depressive symptoms (score 5 to 9); moderate depressive symptoms (score 10 to 14); moderate-severe depressive symptoms (score 15 to 19); and severe depressive symptoms (score 20 and above) [20]. For this study, scores of ≥15 were used to define the presence of depressive symptoms, as this cut-off point presents better values of sensitivity (68%) and specificity (95%) [20]. In Peru, no studies have been conducted on the sensitivity and specificity of PHQ-9. Scores ≥15 were used to define the presence of depressive symptoms, since it is an indicator of the presence of a major depressive episode that requires treatment [20,21].

The age-standardized prevalence of depressive symptoms was estimated using the World Health Organization (WHO) population as the reference population [22]. The age-standardized prevalence is carried out since it allows us to compare our results with other studies carried out in other countries.

Depressive symptomatology in the last year

A second version of PHQ-9 was also used in ENDES 2014-2018, to evaluate depressive symptoms experienced at some point in the past 12 months. Participants were asked to remember an event in the last 12 months in which they had discomfort or problems. Once the participants identified that troublesome or annoying event, the PHQ-9 was applied based on the two weeks around that event. The definition of depressive symptoms experienced some time in the last year was based on two criteria: 1) a score of ≥15 in the two-week version of PHQ-9, or 2) a score of ≥15 in the modified version of PHQ-9 for the last year. The age-standardized prevalence was also analyzed.

For peer review only

Treatment of depressive symptomatology

A participant was considered to receive treatment for symptoms of depression based on two criteria: 1) depressive symptoms had been recorded in the previous year or the past two weeks (PHQ-9 ≥ 15), and 2) they had self-reported receiving treatment for depression from a health professional in the last 12 months. There were three response options ("yes", "no", or "I do not remember"). Participants were only considered having received treatment if an affirmative answer was provided. It should be noted that ENDES data did not include information about the frequency or type of treatment received.

Other variables

In addition to the previous variables mentioned, a set of sociodemographic variables were also taken into consideration. Socioeconomic status (SES) was defined in quintiles (very low, low, middle, high, very high) based on a wealth index available in the ENDES [23]. This index is calculated using the assets and services the participants report having in their house. The calculation of this index can be found in Rutstein and Johnson [23]. As a continuous variable, age was split into four groups (15 to 34, 35 to 54, 55 to 74, and 75 to more). The sex (male vs. female), the study area (urban/rural), and the year of ENDES evaluation were also taken into consideration.

Statistical methods

All statistical analyses were performed using Stata 13 (StataCorp, College Station, TX, USA) and the graphics were elaborated using the ggplot libraries in R (version 3. 5. 1) and QGIS v2.18. The number and percentage of participants excluded from analyses were recorded (see Supplement 1). A descriptive analysis of the participants was carried out for each year of ENDES. An analysis was conducted on the subgroup of participants reporting depressive symptoms over the past year in order to determine the proportion of those who reported receiving treatment. The "svy subpop" command of STATA was used to perform the analysis of subgroups.

Analysis was conducted at both country and regional levels. A 95% confidence interval (95% CI) was calculated for the prevalence at the regional and national level. The Mann-Kendall Tau test was used to evaluate a possible trend in proportions of the population. For the comparisons between groups, the Pearson χ^2 test was used to compare those receiving treatment and the sociodemographic characteristics in people with depressive symptoms. Crude and adjusted models were created using a generalized linear model, assuming a Poisson distribution, link log and robust variance was used as suggested in the literature [24]. Prevalence ratios (PR) and 95% CI were reported. All the analyses performed took into consideration the complex sampling design of the study.

An additional post hoc analysis was performed, which conditions that participants with depressive symptoms must meet the DSM-V criteria. For this sub-analysis participants must have feelings of sadness or anhedonia (have a score of one on items 1 or 2 of the PHQ-9) and at least five of the other seven indicators (have a score of at least one on five of the other seven items of the PHQ-9).

Ethics

The data used in our study are openly accessible to the general public and does not use any personally identifiable information (anonymous), and consequently does not represent an ethical risk for participants. The National Institute of Statistics, a Peruvian government organization, was responsible for the collection of ENDES data. This institution requested the consent of participants in order to obtain the information required in the survey. For each person of legal age (18 years old and above), the informed consent for the collection of information was taken. In the case of minors (17 years old and below) the request for consent was read to one of their parents or to the adult responsible for the minor in order to allow for evaluation of the minor.

For peer review only

Patient and Public Involvement

The patients or members of the public were not involved in the design, or conduct, or reporting, or dissemination plans of the research.

RESULTS

Participants

The ENDES between 2014 and 2018 included the data of 166,290 participants. After excluding all records with incomplete information (n = 5,229, see Supplement 1), a total of 161,061 participants was included. Of these, 51.7% were women, the mean age was 40.3 years (SD = 17.1), and 73% lived in urban settings. The sociodemographic characteristics of the participants for each year are shown in Table 1.

Prevalence of depressive symptomatology

In Peru, between 2014 and 2018, the age-standardized prevalence of depressive symptoms in the last two weeks (p = 0.61) and in the last year (p = 0.46) did not show a significant trend. In 2018, the age-standardized prevalence in the last two weeks and in the last year was 2.3% (n=892, 95% CI: 2.1%−2.5%) and 6.2% (n=2,291, 95% CI: 5.8%−6.6%), respectively (See Figure 1A). On the other hand, in 2018, the un-standardized prevalence of depressive symptoms in the last two weeks was 2.7% (95% CI: 2.4%-2.9%) and in the last year was 6.2% (95% CI: 5.8%−6.6%) and therefore showed little to no difference from the age-standardized prevalence results.

Women had a higher age-standardized prevalence of depressive symptoms in the last two weeks and in the last year, in comparison to men. Adults older than 75 years and individuals in the lowest SES quintile were those with the highest age-standardized prevalence in the last two weeks and in the last year (see Table 2). Figure 2 shows the age-standardized prevalence of depressive symptoms by region. There was no variation in the trends over time in any of the evaluated regions.

When performing the subanalysis that includes the DSM-V criteria, the trend results are not modified.

Treatment of depressive symptomatology

At the national level, the proportion of people with depressive symptoms in the last year who reported receiving any treatment did not show any significant changes over time (p = 0.82 for trends, see figure 1B). In 2018, 14.4% (n= 292, 95% CI: 12.2%−16.9%) of people with depressive symptoms in the last year reported having received some type of treatment from a healthcare professional. A higher percentage of women self-reporting for treatment was found when compared to men (only 2018, 15.1% in women and 13.3% in men, p=0.42), however it was not found to be statistically significant.

The individuals in the highest SES quintile (26.1% in 2018) have a much higher percentage of treatment than those in the lowest SES quintile (6.2% in 2018). The probability of receiving treatment was almost five times greater in the highest SES quintile compared to the lowest SES quintile (RP=5.82, 95% IC: 2.54−10.18, see Supplement 2). No trend of sociodemographic characteristics between 2014 and 2018 was found. At the regional level, the highest percentage of people receiving treatment was on the coast (see figure 2C). The regions with the highest percentage of treatment were Callao (38.5%) and La Libertad (28.8%) and those with the lowest percentage of treatment were Puno (4.8%) and Huancavelica (5.5%), which are regions both in located the highlands (see Supplement 3).

For peer review only

When performing the subanalysis that includes the DSM-V criteria, the trend results are not modified.

DISCUSSION

Main results

In Peru, from 2014 to 2018, no changes in trends (in the two weeks prior and over the last year of the latest ENDES evaluation) were found in the age-standardized prevalence of symptoms of depression and in the percentage of people reporting having received treatment to improve these symptoms. The proportion of people with depressive symptoms receiving treatment was lower in people who live in rural areas and who are of a low socioeconomic level.

The results from this study therefore provide evidence for a need of an increased commitment and focus on the mental health reforms recently initiated by the Peruvian Ministry of Health. Despite strong evidence of a high prevalence of depression in the country, treatment rates still remain low. This, along with significant socio-economic inequalities across the country requires an increased allocation of funds and additional resources in order to prevent and treat depression in the population.

Comparison with other studies

Prevalence of depressive symptomatology

When compared to other countries in Latin America, Peru has demonstrated a lower (un-standardized) prevalence of depressive symptoms than Brazil (9.7%) whilst sharing similar levels to those found in Colombia (5.1%) [25 26]. These findings have additionally been supported by other studies [1]. Contrastingly, high-income countries such as Australia (9.8%) and the United States (8.4%) have exhibited higher age-standardized prevalences than those found in Peru [27 28]. Other countries, have also reported that high-income countries, such as the USA, Brazil, and Australia, have a population with a higher proportion of people suffering from non-communicable and chronic diseases. There is evidence that people with chronic and non-communicable diseases have a higher proportion of depressive symptoms [29], hence the higher prevalence estimate in high-income countries.

When analyzing the differences between groups, it was identified that women have a higher age-standardized prevalence. Sex is a factor that could be influencing the difference between groups because women tend to have an increased risk of having depressive symptoms [5]. This may be because of cultural factors, the women are more likely to provide more information about their feelings during the evaluation whilst men may not report feelings of sadness due to cultural factors associated with gender. Another possible reason could be for biological factors, the women have more estrogen and experience more abrupt hormonal changes than men (menstruation, pregnancy, and menopause). However, this last reason is a very limited explanation to explain such a complex phenomenon [30]. However, this last reason is a very limited explanation to explain such a complex phenomenon.

No variation in the prevalence of depressive symptoms was identified in Peru between 2014 and 2018. Our results are consistent with a meta-analysis of 116 epidemiological studies (1990 to 2010) [9] and with other epidemiological studies with only two measurements over several years conducted in Chile (2003 vs. 2010) and Germany (1997–1999 vs. 2009–2012) [31 32]. Contrastingly, two population-based studies found that there has been an increase in depressive symptoms. The first was carried out in the USA that measured prevalence over a period of 11 years (2005 to 2015) and the second was conducted over the course of two years in Denmark (2000 vs. 2006) [12 13]. The heterogeneity presented in these results can be explained by the variation in the number of measurements and the amount of time evaluated between the different studies. In order to

For peer review only

obtain a stable trend, it is necessary to have consecutive measurements. Although our work only analyzed five-year trends, it is perhaps the first to report estimates across consecutive years using nationally-representative surveys.

Treatment of depressive symptomatology

The treatment gap reported by the Peruvian Ministry of Health (85.9% in 2017) is consistent with our results [33]. However, the treatment gap is greater than those reported in other countries such as USA (72.4%) [34] or Chile (78.8%) [31]. This larger gap could be explained by the stigma associated with seeking mental health care and treatment, preventing patients to actually seek professional attention [35]. Limited resources and lack of trained health personnel could also play a role [33 36].

No significant variation was found in the percentage of people with depressive symptoms receiving treatment in Peru. This situation is different from that of USA [37] and the Republic of Georgia [38], where there has been a significant increase in the proportion of people with depressive symptoms receiving treatment. The difference could be attributed to the new mental health policies that these countries have implemented and the amount of money they invest in mental healthcare. Despite the fact that Peru is undergoing a mental health reform focused on primary care with the implementation of Community Mental Health Centers (CMHCs) [16], the effects at the population level, especially with regards to the proportion of the population with depressive symptoms receiving treatment has not yet been recorded. This could be because it is too early to observe the effects of the CMHCs, as this policy started in 2015 with 22 CMHCs whereas by the end of 2018 there were 106 CMHCs throughout Peru. In addition, CMHCs are not distributed in a decentralized manner. In the region of Lima there are the only three specialized psychiatric hospitals in Peru and 20% of all CMHCs. This could explain why the Callao region (with only 147 km² of surface and located very close to Lima) has the highest percentage of treatment (23.8%), because this region despite its small size has 3 CMHCs, and is very close to the three psychiatric hospitals of Peru and other CMHCs. In comparison with Puno, which has the lowest percentage of treatment (4.8%), possibly due to the fact that it has only 2 CMHCs to supply the territory of 66,997 km². Although largely speculative, these data suggest that the treatment gap could be reduced if CMHCs and other mental health services were available in other regions.

Prevalence and treatment of depressive symptomatology

It could be argued that increasing the percentage of people receiving treatment can help reduce the prevalence of depressive symptoms, but this does not seem to be supported by the evidence. A study conducted in four high-income countries identified that a higher proportion of care is not related to reducing the prevalence of mental health problems [39]. On the other hand, a review suggests that the prevalence of depressive symptoms is reduced if the quality of mental health care is improved, waitlists are erradicated, antidepressants are prescribed with greater caution or psychological treatments are opted for, and preventive interventions linked to the community are carried out [40].

Relevance in public health

The prevalence of depressive symptoms in the last year is very high in Peru; however, only 14.1% of first-level care centers provide mental health services [33]. This situation represents a public health concern as the lack of access to treatment is considered one of the causes of the great treatment gap of mental health experienced by the Peruvian health system [36]. The Peruvian mental health reform seeks, among other objectives, to improve access to mental health care. Nonetheless, our results indicate that it is still too early to see the effects of this reform, since the treatment gap has not been reduced at the population level in recent years (between 2014 to 2018), at least not in people with depressive symptoms. Despite this, the implementation of CMHCs seems to be a very useful policy since the health networks that implemented a CMHC

increased up to four times the number of mental health care consultations, compared to before its implementation [33]. Based on this, we could expect that the implementation of new CMHCs might increase the number of those suffering from mental health problems being attended to, and thereby reduce the treatment gap.
There is evidence that people with a diagnosis of depression are more likely to receive treatment [41], and the implementation of screening for mental health problems in primary care has proven to be very useful in other countries to improve the diagnosis and follow-up [42] [43]. However, population-based screening for depressive symptoms –or other mental health problems– has not been implemented in any Peruvian clinical guidelines [44] [45]. Even though CMHCs aim to screen for mental health problems, their capacity is still limited; thus, it is not feasible to cover large populations. Even if this screening initiative was conducted by other primary care facilities, there is a limited number of psychologists, psychiatrists, and primary care physicians who can perform these screening tests [36]. Therefore, should a population-based screening plan be implemented, it would be necessary to involve lay people (e.g., community health workers) to fill the gap in trained health personnel. Finally, for a clinical practice guideline to be successfully implemented and accepted by the patients, they themselves should be involved in the development of these guidelines [46].

Strengths and limitations
The present study includes the analysis of five years of information from nationally-representative surveys of the Peruvian population and it is the first study assessing the trends of depressive symptoms in Peru. However, this study has limitations. First, the period evaluated was short (five years). If prevalence or treatment were to increase, this increase would most likely be small but sustained annually, so it would be necessary to have a greater number of measurements (10 or 20 years) in order to be able to identify a trend of higher or a decrease of depression symptoms. Second, although the evaluation of depressive symptoms was performed using a valid tool [47], this does not replace an evaluation conducted by a psychiatrist; thus, misclassification may be an issue. However, any misclassification present in the study may be non-differential because the same methods were used to evaluate each year and in each group. Third, another element that could generate a misclassification is the cut-off point used to classify people with depressive symptoms (PHQ-9's scores ≥15). There is evidence suggesting the use of the cut-off point of 15 in PHQ-9 since the severity of depressive symptoms is consistent with a greater depression that normally requires medication [19] [20]. In posthoc analysis, when using the PHQ-9 cutoff of 10, no changes were seen in trends in prevalence and treatment. Finally, other important variables that would have allowed a better understanding of the results were not included, for example, type of treatment received, duration of treatment, and if the participant has previously received a diagnosis of depression.

Conclusions
No significant trend was found in the age-standardized prevalence of depressive symptoms (in the last two weeks and in the last year) or in the percentage of people receiving treatment. The percentage of treatment was lower in people with depressive symptoms who lived in rural areas and who were of a low socioeconomic level. It is necessary to implement policies and interventions to reduce the treatment gap and the prevalence of depressive symptoms accounting for social innequalities across the country.

CONTRIBUTORSHIP STATEMENT
David Villarreal-Zegarra: Conceived and designed the analysis; collected the data; contributed data or analysis tools; performed the analysis; wrote the paper.

For peer review only

Milagros Cabrera-Alva: Contributed data or analysis tools; wrote the paper.

Rodrigo Carrillo-Larco: Conceived and designed the analysis; contributed data or analysis tools; performed the analysis; wrote the paper; supervision of the paper.

Antonio Bernabe-Ortiz: Conceived and designed the analysis; contributed data or analysis tools; performed the analysis; wrote the paper; supervision of the paper.

COMPETING INTERESTS

The authors have declared that no competing interests exist.

FUNDING

This work was supported by the Strategic Award, Wellcome Trust-Imperial College Centre for Global Health Research (100693/Z/12/Z), Imperial College London Wellcome Trust Institutional Strategic Support Fund [Global Health Clinical Research Training Fellowship] (294834/Z/16/Z ISSF ICL), and Rodrigo M Carrillo-Larco is supported by a Wellcome Trust International Training Fellowship (214185/Z/18/Z). The funders had no role in study design, data collection and analysis, decision to publish, or preparation of the manuscript.

GRANT DISCLOSURES

The following grant information was disclosed by the authors:

Strategic Award, Wellcome Trust-Imperial College Centre for Global Health Research: 100693/Z/12/Z.

Imperial College London Wellcome Trust Institutional Strategic Support Fund: 294834/Z/16/Z ISSF ICL.

Wellcome Trust International Training Fellowship: 214185/Z/18/Z.

DATA SHARING STATEMENT

The database is freely accessible from the website of the National Institute of Statistics of Peru, URL: http://iinei.inei.gob.pe/microdatos/

The information can be obtained by entering the survey query tab and selecting the ENDES data using the health module data. Only cross-sectional information from 2014 to 2018 for the ENDES Health Questionnaire was used.

ACKNOWLEDGEMENTS

DVZ thanks to Helena Kesar and Qory Mosoco for his style review in the final version of the English manuscript.

ABBREVIATIONS

CMHCs: Community Mental Health Centers.
DSM-IV: Diagnostic and Statistical Manual of Mental Disorders, 4th Edition.
ENDES: Demographic and Health Survey of Peru.
SD: Standard Deviation.
SES: Socioeconomic Status.
PHQ-9: Patient's Health Questionnaire.
PR: Prevalence Ratios.
USA: United States of America.

For peer review only

WHO: World Health Organization.
95%CI: 95% confidence interval.

REFERENCES

1. World Health Organization. Depression and Other Common Mental Disorders. Global Health Estimates. Geneve: World Health Organization 2017.
2. Hsieh CR, Qin X. Depression hurts, depression costs: The medical spending attributable to depression and depressive symptoms in China. *Health economics* 2018;27(3):525-44. doi: 10.1002/hec.3604 [published Online First: 2017/10/11]
3. Vasiliadis HM, Dezetter A, Latimer E, et al. Assessing the Costs and Benefits of Insuring Psychological Services as Part of Medicare for Depression in Canada. *Psychiatric services* 2017;68(9):899-906. doi: 10.1176/appi.ps.201600395 [published Online First: 2017/05/16]
4. World Health Organization. The global burden of disease: 2004 update. Geneva: WHO 2008.
5. Salk RH, Hyde JS, Abramson LY. Gender differences in depression in representative national samples: Meta-analyses of diagnoses and symptoms. *Psychol Bull* 2017;143(8):783-822. doi: 10.1037/bul0000102 [published Online First: 2017/04/28]
6. Shao J, Li D, Zhang D, et al. Birth cohort changes in the depressive symptoms of Chinese older adults: a cross-temporal meta-analysis. *International Journal of Geriatric Psychiatry* 2013;28(11):1101-08. doi: doi:10.1002/gps.3942
7. Lee E-W, Park J-H. Individual and Socioeconomic Contextual Effects on Depressive Symptom in Korea: Multilevel Analysis of Cross-sectional Nationwide Survey. *Journal of Korean medical science* 2015;30(2):186-93.
8. Eaton J, McCay L, Semrau M, et al. Scale up of services for mental health in low-income and middle-income countries. *Lancet (London, England)* 2011;378(9802):1592-603. doi: 10.1016/s0140-6736(11)60891-x [published Online First: 2011/10/20]
9. Baxter AJ, Scott KM, Ferrari AJ, et al. Challenging the myth of an "epidemic" of common mental disorders: trends in the global prevalence of anxiety and depression between 1990 and 2010. *Depression and Anxiety* 2014;31(6):506-16. doi: doi:10.1002/da.22230
10. GBD 2016 Disease and Injury Incidence and Prevalence Collaborators. Global, regional, and national incidence, prevalence, and years lived with disability for 328 diseases and injuries for 195 countries, 1990-2016: a systematic analysis for the Global Burden of Disease Study 2016. *Lancet (London, England)* 2017;390(10100):1211-59. doi: 10.1016/s0140-6736(17)32154-2 [published Online First: 2017/09/19]
11. GBD 2015 Disease and Injury Incidence and Prevalence Collaborators. Global, regional, and national incidence, prevalence, and years lived with disability for 310 diseases and injuries, 1990-2015: a systematic analysis for the Global Burden of Disease Study 2015. *The Lancet* 2016;388(10053):1545-602. doi: 10.1016/s0140-6736(16)31678-6 [published Online First: 2016/10/14]
12. Andersen I, Thielen K, Bech P, et al. Increasing prevalence of depression from 2000 to 2006. *Scandinavian Journal of Public Health* 2011;39(8):857-63. doi: 10.1177/1403494811424611
13. Weinberger AH, Gbedemah M, Martinez AM, et al. Trends in depression prevalence in the USA from 2005 to 2015: widening disparities in vulnerable groups. *Psychological medicine* 2018;48(8):1308-15. doi: 10.1017/s0033291717002781 [published Online First: 2017/10/13]
14. Guerra M, Prina AM, Ferri CP, et al. A comparative cross-cultural study of the prevalence of late life depression in low and middle income countries. *Journal of Affective Disorders* 2016;190:362-68. doi: https://doi.org/10.1016/j.jad.2015.09.004

For peer review only

15. Thornicroft G, Chatterji S, Evans-Lacko S, et al. Undertreatment of people with major depressive disorder in 21 countries. *British Journal of Psychiatry* 2018;210(2):119-24. doi: 10.1192/bjp.bp.116.188078 [published Online First: 01/02]
16. Toyama M, Castillo H, Galea JT, et al. Peruvian Mental Health Reform: A Framework for Scaling-up Mental Health Services. *International journal of health policy and management* 2017;6(9):501-08. doi: 10.15171/ijhpm.2017.07 [published Online First: 2017/09/28]
17. Instituto Nacional de Estadística e Informática. Perú: encuesta demográfica y de salud familiar ENDES 2014. Perú: Instituto Nacional de Estadística e Informática 2015.
18. Instituto Nacional de Estadística e Informática. Perú - Encuesta Demográfica y de Salud Familiar 2017 Peru: Instituto Nacional de Estadística e Informática; 2018 [Available from: https://webinei.inei.gob.pe/anda_inei/index.php/catalog/649#page=sampling&tab=related-materials accessed December 2019 2019.
19. Villarreal-Zegarra D, Copez-Lonzoy A, Bernabe-Ortiz A, et al. Valid group comparisons can be made with the Patient Health Questionnaire (PHQ-9): A measurement invariance study across groups by demographic characteristics. *PloS one* 2019;14(9):e0221717. doi: 10.1371/journal.pone.0221717 [published Online First: 2019/09/10]
20. Kroenke K, Spitzer RL, Williams JB. The PHQ-9: validity of a brief depression severity measure. *J Gen Intern Med* 2001;16(9):606-13. [published Online First: 2001/09/15]
21. Manea L, Gilbody S, McMillan D. A diagnostic meta-analysis of the Patient Health Questionnaire-9 (PHQ-9) algorithm scoring method as a screen for depression. *General hospital psychiatry* 2015;37(1):67-75. doi: 10.1016/j.genhosppsych.2014.09.009 [published Online First: 2014/12/03]
22. Ahmad OB, Boschi-Pinto C, Lopez AD, et al. Age standardization of rates: a new who standard. World Health Organization: Geneva 2001.
23. Rutstein SO, Johnson K. The DHS wealth index. DHS comparative reports no. 6. Calverton, Maryland: ORC Macro 2004.
24. Barros AJ, Hirakata VN. Alternatives for logistic regression in cross-sectional studies: an empirical comparison of models that directly estimate the prevalence ratio. *BMC Med Res Methodol* 2003;3(1):21.
25. Gómez-Restrepo C, Tamayo Martínez N, Bohórquez A, et al. Trastornos depresivos y de ansiedad y factores asociados en la población adulta colombiana, Encuesta Nacional de Salud Mental 2015. *Revista Colombiana de Psiquiatría* 2016;45:58-67. doi: https://doi.org/10.1016/j.rcp.2016.04.009
26. Barros MBA, Lima MG, Azevedo RCS, et al. Depression and health behaviors in Brazilian adults - PNS 2013. *Rev Saude Publica* 2017;51(suppl 1):8s. doi: 10.1590/s1518-8787.2017051000084 [published Online First: 2017/06/08]
27. Zhao G, Ford ES, Dhingra S, et al. Depression and anxiety among US adults: associations with body mass index. *International journal of obesity (2005)* 2009;33(2):257-66. doi: 10.1038/ijo.2008.268 [published Online First: 2009/01/07]
28. Atlantis E, Goldney RD, Eckert KA, et al. Trends in health-related quality of life and health service use associated with comorbid diabetes and major depression in South Australia, 1998-2008. *Social psychiatry and psychiatric epidemiology* 2012;47(6):871-7. doi: 10.1007/s00127-011-0394-4 [published Online First: 2011/05/19]
29. DeJean D, Giacomini M, Vanstone M, et al. Patient experiences of depression and anxiety with chronic disease: a systematic review and qualitative meta-synthesis. *Ontario health technology assessment series* 2013;13(16):1-33. [published Online First: 2013/11/15]
30. Albert PR. Why is depression more prevalent in women? *Journal of psychiatry & neuroscience : JPN* 2015;40(4):219-21. doi: 10.1503/jpn.150205

For peer review only

31. Markkula N, Zitko P, Pena S, et al. Prevalence, trends, correlates and treatment of depression in Chile in 2003 to 2010. *Social psychiatry and psychiatric epidemiology* 2017;52(4):399-409. doi: 10.1007/s00127-017-1346-4 [published Online First: 2017/02/16]
32. Bretschneider J, Janitza S, Jacobi F, et al. Time trends in depression prevalence and health-related correlates: results from population-based surveys in Germany 1997–1999 vs. 2009–2012. *BMC psychiatry* 2018;18(1):394. doi: 10.1186/s12888-018-1973-7
33. Ministerio de Salud. Plan Nacional de Fortalecimiento de Servicios de Salud Mental Comunitaria 2018 – 2021. Perú: Ministerio de Salud 2018.
34. Wittayanukorn S, Qian J, Hansen RA. Prevalence of depressive symptoms and predictors of treatment among U.S. adults from 2005 to 2010. *Gen Hosp Psychiatry* 2014;36(3):330-6. doi: 10.1016/j.genhosppsych.2013.12.009 [published Online First: 2014/01/28]
35. Pattyn E, Verhaeghe M, Sercu C, et al. Public stigma and self-stigma: differential association with attitudes toward formal and informal help seeking. *Psychiatric services (Washington, DC)* 2014;65(2):232-8. doi: 10.1176/appi.ps.201200561 [published Online First: 2013/11/16]
36. Grupo del Banco Mundial. Movimiento por el cuidado comunitario de la salud mental en el Perú. Lima: Grupo del Banco Mundial 2019.
37. Mojtabai R, Olfson M, Han B. National Trends in the Prevalence and Treatment of Depression in Adolescents and Young Adults. *Pediatrics* 2016;138(6) doi: 10.1542/peds.2016-1878 [published Online First: 2016/12/13]
38. Kenchadze V, Chkonia E, Beria Z. Trends in the use of outpatient psychiatric services in the republic of Georgia. *Georgian medical news* 2013(215):60-5. [published Online First: 2013/03/14]
39. Jorm AF, Patten SB, Brugha TS, et al. Has increased provision of treatment reduced the prevalence of common mental disorders? Review of the evidence from four countries. *World psychiatry : official journal of the World Psychiatric Association (WPA)* 2017;16(1):90-99. doi: 10.1002/wps.20388 [published Online First: 2017/01/28]
40. Ormel J, Kessler RC, Schoevers R. Depression: more treatment but no drop in prevalence: how effective is treatment? And can we do better? *Current opinion in psychiatry* 2019;32(4):348-54. doi: 10.1097/yco.0000000000000505 [published Online First: 2019/03/12]
41. Fujii RK, Goren A, Annunziata K, et al. Prevalence, Awareness, Treatment, and Burden of Major Depressive Disorder: Estimates from the National Health and Wellness Survey in Brazil. *Value in health regional issues* 2012;1(2):235-43. doi: 10.1016/j.vhri.2012.09.011 [published Online First: 2012/12/01]
42. Siu AL, Bibbins-Domingo K, Grossman DC, et al. Screening for Depression in Adults: US Preventive Services Task Force Recommendation Statement. *Jama* 2016;315(4):380-7. doi: 10.1001/jama.2015.18392 [published Online First: 2016/01/28]
43. Vilagut G, Forero CG, Barbaglia G, et al. Screening for Depression in the General Population with the Center for Epidemiologic Studies Depression (CES-D): A Systematic Review with Meta-Analysis. *PloS one* 2016;11(5):e0155431. doi: 10.1371/journal.pone.0155431 [published Online First: 2016/05/18]
44. Ministerio de Salud. Guía de practica clínica en depresión. Perú: Ministerio de Salud 2008.
45. Instituto Nacional de Salud. Guía de práctica clínica para el tratamiento de depresión en adultos - versión extensa. Lima: Instituto Nacional de Salud 2017.
46. Zafra-Tanaka JH, Goicochea-Lugo S, Villarreal-Zegarra D, et al. Characteristics and quality of clinical practice guidelines for depression in adults: a scoping review. *BMC psychiatry* 2019;19(1):76. doi: 10.1186/s12888-019-2057-z

For peer review only

47. Baader M T, Molina F JL, Venezian B S, et al. Validación y utilidad de la encuesta PHQ-9 (Patient Health Questionnaire) en el diagnóstico de depresión en pacientes usuarios de atención primaria en Chile. *Rev Chil Neuropsiquiatr* 2012;50:10-22.

For peer review only

FIGURES AND TABLES

Figure 1. Age-standardized prevalence of depressive symptoms and percentage of treatment in Peru between 2014 to 2018.
A) Age-standardized prevalence of depressive symptoms in the last year and the last two weeks in Peru by year. B) Percentage of people with depressive symptoms receiving treatment in Peru by year. All the analyzes the weighted proportion by complex sampling was used.

Figure 2. Age-standardized prevalence of depressive symptoms and percentage of treatment in Peru by region in 2018.
A) Age-standardized prevalence of depressive symptoms in the last two week from Peru for region in 2018. B) Age-standardized prevalence of depressive symptoms in the last year in Peru for region in 2018. C) Percentage of people with depressive symptoms in the last year and receiving treatment from Peru for region in 2018. All the analyzes the weighted proportion by complex sampling was used.
Figure designed by the authors.

For peer review only

Table 1. Descriptive characteristics of the participants included in the study by year.

		2014 (n=27,633)		2015 (n=33,573)		2016 (n=32,373)		2017 (n=33,794)		2018 (n=34,476)	
		n	%	*n*	%	*n*	%	*n*	%	*n*	%
Sex	Male	12,806	46.3%	14,788	48.9%	14,126	48.9%	14,424	48.5%	14,696	48.4%
	Female	14,827	53.7%	18,573	51.1%	18,247	51.1%	18,794	51.5%	19,780	51.6%
Age	15-34	11,022	43.1%	16,576	43.1%	15,498	44.4%	15,913	43.6%	16,121	42.7%
	35-54	9,395	33.9%	10,887	33.9%	10,740	33.8%	10,819	34.5%	11,692	34.6%
	55-74	5,460	18.0%	4,685	18.0%	4,791	16.8%	5,030	17.0%	5,332	17.7%
	75+	1,756	5.0%	1,213	5.0%	1,344	5.0%	1,456	4.9%	1,331	5.0%
Area	Rural	10,663	24.9%	11,453	34.5%	11,113	35.2%	11,349	20.8%	11,923	19.6%
	Urban	16,970	75.1%	21,908	65.5%	21,260	64.8%	21,869	79.3%	22,553	80.4%
SES	Very low	8,151	18.7%	9,370	26.3%	8,967	26.4%	10,045	18.5%	11,019	18.7%
	Low	6,782	19.2%	8,376	21.2%	8,464	22.0%	8,575	20.8%	8,514	20.6%
	Middle	5,153	19.9%	6,459	18.3%	6,361	18.5%	6,368	21.0%	6,379	20.9%
	High	4,103	21.0%	5,089	17.7%	5,056	17.4%	4,846	20.3%	4,909	20.4%
	Very high	3,444	21.2%	4,067	16.4%	3,525	15.8%	3,384	19.4%	3,655	19.4%

Note: The weighted proportion by complex sampling. SES = Socioeconomic status.

For peer review only

Table 2. Age-standardized prevalence of depressive symptoms and percentage of treatment in Peru by selected socio-demographic characteristics (2014-2018, with cut-off point 15).

			2014	2015	2016	2017	2018
Age-standardized prevalence of Depressive symptoms in the last two week †	Sex	Male	1.5%	1.9%	1.9%	1.3%	1.1%
		Female	3.6%	3.9%	4.0%	3.3%	3.4%
	Age	15-34	1.5%	1.5%	1.6%	1.2%	1.1%
		35-54	2.9%	2.6%	2.6%	2.5%	2.3%
		55-74	4.0%	5.7%	5.0%	4.1%	4.1%
		75+	6.7%	9.0%	11.2%	6.0%	6.5%
	Area	Rural	2.9%	3.5%	3.4%	3.2%	3.3%
		Urban	2.5%	2.5%	2.7%	2.1%	2.0%
	SES	Very low	3.0%	3.8%	3.6%	3.2%	3.5%
		Low	3.1%	3.3%	2.8%	3.1%	2.7%
		Middle	3.3%	3.2%	3.5%	2.8%	2.4%
		High	2.5%	2.1%	2.6%	1.6%	1.7%
		Very high	1.6%	1.7%	1.7%	1.2%	1.2%
Age-standardized prevalence of Depressive symptoms in the last year †	Sex	Male	3.7%	4.3%	4.0%	3.5%	3.9%
		Female	8.5%	9.1%	9.3%	8.2%	8.2%
	Age	15-34	4.5%	4.4%	4.6%	4.1%	4.3%
		35-54	6.9%	6.7%	6.5%	6.0%	6.1%
		55-74	8.1%	10.6%	9.7%	9.3%	9.0%
		75+	12.5%	15.3%	17.3%	9.6%	13.2%
	Area	Rural	7.2%	7.9%	7.6%	7.4%	8.1%
		Urban	5.9%	6.0%	6.1%	5.5%	5.6%
	SES	Very low	7.4%	8.1%	7.8%	7.3%	8.4%
		Low	7.4%	7.7%	7.1%	7.4%	7.1%
		Middle	7.1%	6.9%	7.4%	6.5%	6.6%
		High	5.4%	5.7%	5.9%	4.6%	5.0%
		Very high	4.8%	4.5%	4.3%	4.3%	4.3%
Percentage of People with depressive symptoms receiving treatment ‡	Sex	Male	13.3%	11.9%	10.7%	10.6%	11.1%
		Female	15.1%	14.7%	16.9%	16.6%	15.9%
	Age	15-34	15.7%	14.8%	19.7%	19.2%	14.7%
		35-54	17.3%	16.1%	18.0%	15.9%	13.6%
		55-74	12.1%	12.2%	10.5%	10.0%	16.0%
		75+	7.4%	8.5%	6.2%	10.0%	12.5%
	Area	Rural	7.9%	7.2%	7.1%	5.6%	7.8%
		Urban	17.5%	18.6%	21.0%	18.4%	16.9%
	SES	Very low	5.8%	5.7%	5.8%	3.3%	6.2%
		Low	8.8%	11.6%	14.1%	13.2%	10.1%
		Middle	16.3%	13.8%	17.1%	15.3%	17.4%
		High	20.3%	22.0%	24.7%	14.2%	20.4%
		Very high	27.1%	31.3%	31.0%	38.4%	26.1%

Note: All the analyzes the weighted proportion by complex sampling was used. SES = Socioeconomic status. † The analysis considered the total of Peruvian population. ‡ An analysis is done by subgroups, considering only people who have depressive symptoms.

For peer review only

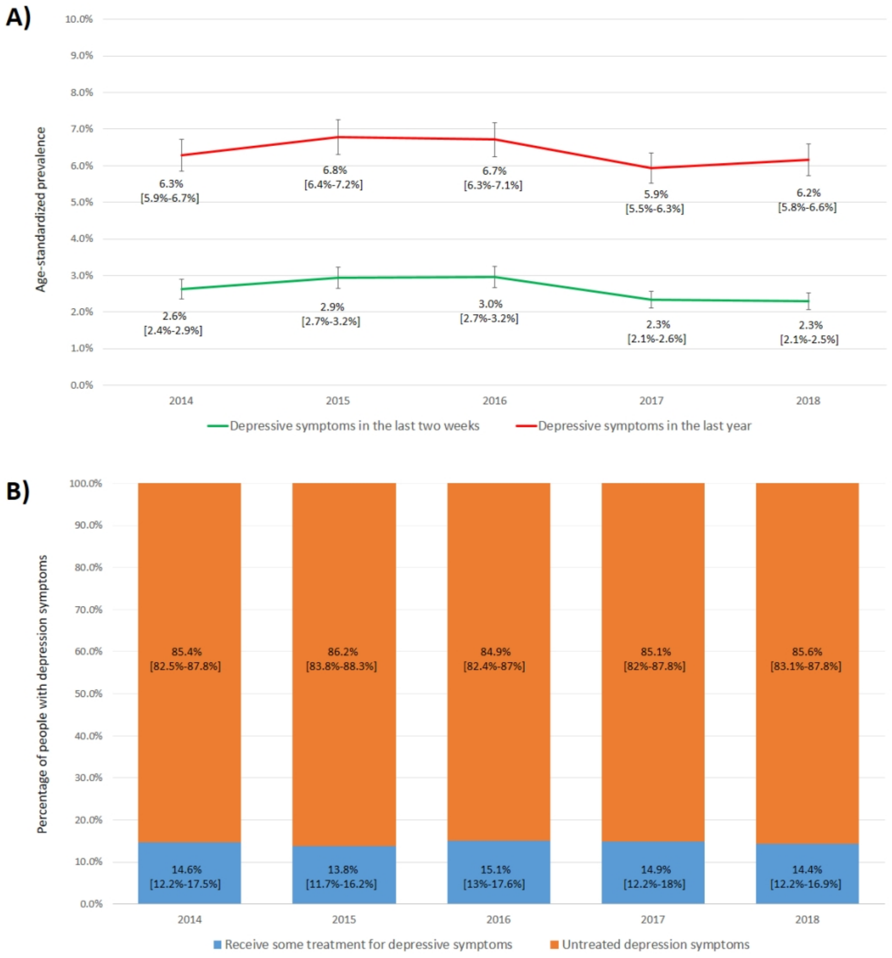

Figure 1. Age-standardized prevalence of depressive symptoms and percentage of treatment in Peru between 2014 to 2018.

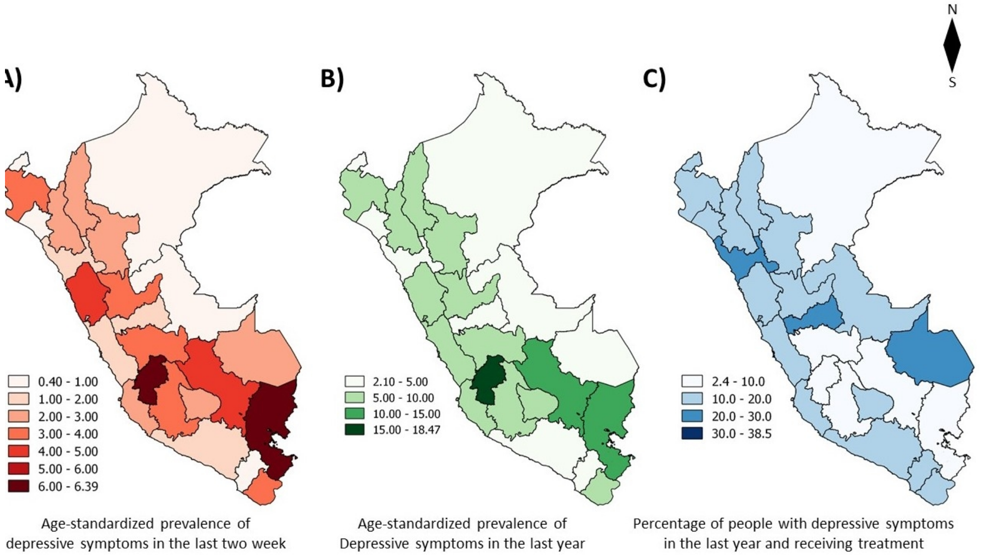

Figure 2. Age-standardized prevalence of depressive symptoms and percentage of treatment in Peru by region in 2018.

344x207mm (96 x 96 DPI)

Supplement 1. Descriptive characteristics of the participants not included in the study (2014-2018).

		2014		2015		2016		2017		2018	
		n	%	*n*	%	*n*	%	*n*	%	*n*	%
Sex	Male	0	0.0%	1	0.1%	0	0.0%	0	0.0%	0	0.00%
	Female	0	0.0%	8	0.7%	0	0.0%	1	0.1%	0	0.00%
	Missing	1,160	100.0%	1,101	99.2%	1,166	100.0%	880	99.9%	912	100.00%
Age	15-34	0	0.0%	4	0.3%	0	0.0%	0	0.0%	0	0.00%
	35-54	0	0.0%	3	0.3%	0	0.0%	0	0.0%	0	0.00%
	55-74	0	0.0%	1	0.1%	0	0.0%	0	0.0%	0	0.00%
	75+	0	0.0%	1	0.1%	0	0.0%	74	0.1%	0	0.00%
	Missing	1,160	100.0%	1,101	99.2%	1,166	100.0%	880	99.9%	912	100.00%
Area	Rural	261	22.5%	289	26.0%	253	21.7%	194	22.0%	175	19.2%
	Urban	899	77.5%	821	74.0%	913	78.3%	687	78.0%	737	80.8%
	Missing	0	0.0%	0	0.0%	0	0.0%	0	0.0%	0	0.00%
SES	Very low	207	17.8%	261	23.5%	214	18.4%	181	20.5%	155	17.0%
	Low	205	17.7%	201	18.1%	209	17.9%	152	17.3%	162	17.8%
	Middle	230	19.8%	192	17.3%	232	19.9%	152	17.3%	199	21.8%
	High	241	20.8%	225	20.3%	258	22.1%	204	23.2%	159	17.4%
	Very high	277	23.9%	231	20.8%	253	21.7%	192	21.8%	237	26.0%
	Missing	0	0.0%	0	0.0%	0	0.0%	0	0.0%	0	0.00%

Note: SES = Socioeconomic status.

For peer review only

Supplement 2. Association between receiving treatment and sociodemographic characteristics in people with depressive symptoms in last year.

		Model crude PR (95% CI)	Adjusted model * PR (95% CI)
Sex	Male	1	1
	Female	1.42 (0.96 - 2.10)	1.30 (0.88 - 1.92)
Age	15-34	1	1
	35-54	0.92 (0.64 - 1.32)	0.98 (0.69 - 1.41)
	55-74	1.09 (0.71 - 1.67)	1.23 (0.81 - 1.87)
	75+	0.85 (0.37 - 1.91)	1.08 (0.49 - 2.39)
Area	Rural	1	1
	Urban	**2.18 (1.58 - 3.00)**	0.81 (0.48 - 1.37)
SES	Very low	1	1
	Low	**1.65 (1.05 - 2.60)**	**1.89 (1.06 - 3.38)**
	Middle	**2.84 (1.78 - 4.50)**	**3.36 (1.73 - 6.53)**
	High	**3.32 (2.04 - 5.40)**	**3.92 (1.96 - 7.87)**
	Very high	**4.25 (2.65 - 6.81)**	**5.08 (2.54 - 10.18)**

Note: Analysis is done by subgroups, considering only people who have depressive symptoms, the complex sampling was used. SES = Socioeconomic status.
* Adjusted by sex, age, socioeconomic status, and area.

For peer review only

Supplement 3. Age-standardized prevalence of depressive symptoms and percentage of treatment in Peru by region in 2018 (with cut-off point 15).

Region	Age-standardized prevalence of Depressive symptoms in the last two week *	Age-standardized prevalence of Depressive symptoms in the last year *	Percentage of People with depressive symptoms receiving treatment **
Amazonas	2.19%	5.63%	11.77%
Ancash	4.03%	9.95%	10.67%
Apurímac	2.52%	7.81%	16.63%
Arequipa	1.20%	4.19%	19.51%
Ayacucho	3.68%	8.93%	8.27%
Cajamarca	2.47%	5.54%	11.48%
Callao	1.70%	4.51%	38.51%
Cusco	4.36%	12.36%	9.74%
Huancavelica	6.10%	18.47%	5.89%
Huánuco	3.62%	7.95%	17.87%
Ica	2.99%	5.78%	15.42%
Junín	3.12%	6.87%	9.79%
La Libertad	1.41%	4.15%	28.52%
Lambayeque	0.40%	2.10%	17.19%
Lima	1.69%	5.40%	15.77%
Loreto	0.80%	2.20%	2.38%
Madre de Dios	2.12%	3.63%	22.03%
Moquegua	0.84%	3.36%	10.07%
Pasco	1.27%	4.61%	24.52%
Piura	3.21%	7.91%	14.08%
Puno	6.39%	13.71%	7.12%
San Martín	2.05%	5.16%	12.92%
Tacna	3.86%	7.21%	12.34%
Tumbes	0.88%	2.52%	13.03%
Ucayali	0.75%	3.39%	14.51%

Note: All the analyzes the weighted proportion by complex sampling was used. * The analysis considered the total of Peruvian population. ** An analysis is done by subgroups, considering only people who have depressive symptoms.

For peer review only

BMJ Open

Trends in the prevalence and treatment of depressive symptoms in Peru: A population-based study

Journal:	*BMJ Open*
Manuscript ID	bmjopen-2020-036777.R1
Article Type:	Original research
Date Submitted by the Author:	06-Apr-2020
Complete List of Authors:	Villarreal-Zegarra, David; Instituto Peruano de Orientación Psicológica; Universidad Peruana Cayetano Heredia, CRONICAS Center of Excellence in Chronic Diseases<br>Cabrera-Alva, Milagros; Instituto Peruano de Orientación Psicológica<br>Carrillo-Larco, Rodrigo; Universidad Peruana Cayetano Heredia, CRONICAS Center of Excellence in Chronic Diseases; Imperial College London School of Public Health, Department of Epidemiology and Biostatistics<br>Bernabe-Ortiz, Antonio; Universidad Peruana Cayetano Heredia, CRONICAS Centre of Excellence in Chronic Diseases; Universidad Científica del Sur
**Primary Subject Heading**:	Mental health
Secondary Subject Heading:	Public health, Epidemiology
Keywords:	Depression & mood disorders < PSYCHIATRY, EPIDEMIOLOGY, PUBLIC HEALTH

SCHOLARONE™
Manuscripts

I, the Submitting Author has the right to grant and does grant on behalf of all authors of the Work (as defined in the below author licence), an exclusive licence and/or a non-exclusive licence for contributions from authors who are: i) UK Crown employees; ii) where BMJ has agreed a CC-BY licence shall apply, and/or iii) in accordance with the terms applicable for US Federal Government officers or employees acting as part of their official duties; on a worldwide, perpetual, irrevocable, royalty-free basis to BMJ Publishing Group Ltd ("BMJ") its licensees and where the relevant Journal is co-owned by BMJ to the co-owners of the Journal, to publish the Work in this journal and any other BMJ products and to exploit all rights, as set out in our licence.

The Submitting Author accepts and understands that any supply made under these terms is made by BMJ to the Submitting Author unless you are acting as an employee on behalf of your employer or a postgraduate student of an affiliated institution which is paying any applicable article publishing charge ("APC") for Open Access articles. Where the Submitting Author wishes to make the Work available on an Open Access basis (and intends to pay the relevant APC), the terms of reuse of such Open Access shall be governed by a Creative Commons licence – details of these licences and which Creative Commons licence will apply to this Work are set out in our licence referred to above.

Other than as permitted in any relevant BMJ Author's Self Archiving Policies, I confirm this Work has not been accepted for publication elsewhere, is not being considered for publication elsewhere and does not duplicate material already published. I confirm all authors consent to publication of this Work and authorise the granting of this licence.

For peer review only

Trends in the prevalence and treatment of depressive symptoms in Peru: A population-based study

Authors
David Villarreal-Zegarra [1, 2]
Milagros Cabrera-Alva [2]
Rodrigo M. Carrillo-Larco [1, 3, 4]
Antonio Bernabe-Ortiz [1, 5]

Affiliation
[1] CRONICAS Center of Excellence in Chronic Diseases, Universidad Peruana Cayetano Heredia, Lima, Peru.
[2] Instituto Peruano de Orientación Psicológica, Lima, Peru.
[3] Department of Epidemiology and Biostatistics, School of Public Health, Imperial College London, London, United Kingdom.
[4] Centro de Estudios de Población, Universidad Católica los Ángeles de Chimbote (ULADECH), Chimbote, Peru.
[5] Universidad Científica del Sur, Lima, Peru.

Corresponding author
David Villarreal-Zegarra
E-mail: davidvillarreal@ipops.pe
Avenida Armendáriz 445, Miraflores, Lima 18, Perú
Telf: (+511) 241-6978

Number of Figures: 2

Number of Tables: 3

Number of Supplements: 3

Number of words: 4660

For peer review only

ARTICLE SUMMARY

Objectives: This study aimed to estimate the trends in the prevalence and treatment of depressive symptoms using nationally representative surveys in Peru from 2014 to 2018.

Design: A secondary analysis was conducted using five consecutive nationally representative surveys conducted from 2014-2018, including a total of 166,290 individuals.

Setting: The study was conducted in Peru.

Participants: The participants in each year of measurement were different, and the sampling used was probabilistic in two stages. Men and women, aged ≥15 years, were included.

Main outcome measures: Two versions of the Patient Health Questionnaire (PHQ-9) that focused on the presence of depressive symptoms in the last two weeks and the last year were used. Scores of ≥15 were used as the cut-off point in both versions of the PHQ-9 to define the presence of depressive symptoms. In addition, the treatment rate was based on the proportion of individuals with depressive symptoms in the last year who self-reported having received specific treatment for these symptoms. The age-standardized prevalence was estimated using the World Health Organization population as the reference population.

Results: A total of 161,061 participants were included. There was no evidence of a change in age-standardized prevalence rates of depressive symptoms at two weeks (2.6% in 2014 to 2.3% in 2018), or in the last year (6.3% in 2014 to 6.2% in 2018). No change was found either in the proportion of depressive cases treated in the last year (14.6% in 2014 to 14.4% in 2018). This latter proportion was lower in people with a low level of wealth.

Conclusions: One in fifty people exhibited depressive symptoms during the last two weeks and three in fifty in the last year. Also, one in seven individuals with depressive symptoms reported receiving treatment. However, there was no evidence of a decreasing or increasing trend.

Keywords: Depression, Epidemiology, Mood disorders, Treatment, Primary care.

For peer review only

STRENGTHS AND LIMITATIONS OF THIS STUDY

- ✓ This analysis included information from five nationally representative surveys of the Peruvian population.
- ✓ Our findings were limited as depression assessment was not used psychiatric evaluations or structured interviews; so, only depressive symptoms could be identified.
- ✓ Only five years were evaluated and perhaps more time may be required to identify a significant trend.

For peer review only

INTRODUCTION

Depression is a significant public health issue that affects approximately 322 million people worldwide [1] and has tremendous social and economic cost implications [2 3]. Based on projections, it is estimated that depression will be the leading cause of years lost to disability (DALY´s) by 2030 [4]. Epidemiological surveillance of the trends in depressive symptoms is of high relevance to public health. As changes in the prevalence and treatment can aid researchers and stakeholders make more informed decisions on how to address this challenge.

The prevalence of depressive symptoms can vary depending on several biological, sociodemographic, or lifestyle factors. For example, women [5], older adults [6], and people with a low socioeconomic status [7] have a higher prevalence of depressive symptoms. On the other hand, the fact that a person with depressive symptoms can access treatment also depends on accessibility to health systems. A systematic review identified that lack of human resources, centralization of the health system, and integration in primary care, are barriers to receiving appropriate treatment of depressive symptoms [8].

Currently, there is no consensus on the trends of the prevalence of depressive symptoms. A meta-analysis of 116 epidemiological investigations during the years 1990 and 2010 did not reveal changes in the prevalence of major depressive disorder [9]. Nevertheless, the Global Burden of Disease Studies assessing trends in previous years [10 11], found a decrease of 4.9% in the age-standardized rate of depressive disorders from 2006 to 2016. On the contrary, other longitudinal studies showed an increase in the prevalence of major depressive disorder [12 13]. These mixed results can be attributed to inter-country variations of several demographic and socioeconomic factors, as well as the different criteria used to define depression in research studies. Due to the lack of global consensus, country-specific estimates and trends are significant and can inform local policies and guidelines.

In Peru, the prevalence of depressive symptoms ranges from 14% in urban areas to 12.5% in rural areas [14]. Whereas the prevalence of major depressive disorders reported in the last year was 2.7%, of which a third receive minimally adequate treatment [15]. Moreover, access to treatment for those suffering from depressive disorders is very limited in low and middle-income countries, with only 1 in every 27 patients with major depressive disorder, managing to receive treatment [15]. Since 2015, Peru has been implementing mental health reforms that focus on primary care and the redistribution of its resources from hospital care to community care centers [16].

It is, therefore, fundamental to have up-to-date metrics on the prevalence and level of treatment coverage of depressive symptoms in order to determine an appropriate response to this disease. This study aims to estimate trends in prevalence and treatment of depressive symptoms using nationally representative surveys in Peru between 2014 and 2018.

METHODS

Study design

Data from the National Demographic and Health Survey of Peru (ENDES – Spanish acronym), which collects information on several variables, including poverty, fertility, violence, and health, was utilized in this study. Before being carried out annually, in 1996 it was the first ENDES that conducted, the second in 2000, and the third in 2004. Since 2013, ENDES began to incorporate questions regarding mental health into its evaluation. However, it initially only used a very small sub-sample that was not representative of the general population. From 2014 onwards, the data collected on mental health was nationally and regional representative. Our study, therefore, decided to use data collected by ENDES between the years 2014 and 2018.

For peer review only - http://bmjopen.bmj.com/site/about/guidelines.xhtml

The data collection, in each of the years, used a face-to-face approach and started in February or March, while the completion ended in December [17,18]. Data collection was face-to-face, with each participant evaluated being interviewed.
The participants from whom the data was being collected from in the annual ENDES evaluations were different. Therefore, the data is evaluated in this study came from five different groups (one from each year of evaluation). So, while there was a chance that a participant would repeat in two different years or that two or more participants from the same household would be evaluated in one year, this probability was minuscule.

Participants
The sampling used was probabilistic in two stages and representative at the national and regional levels. The sampling frame in the first stage was the selection of primary sampling units (clusters) based on information from the last census conducted in Peru. In the second stage, the selection of secondary sampling units (households) was carried out based on the information from the cartographic updates and the register of buildings and households made previously [17].
In rural areas, the primary sampling units were comprised of groups ranging from 500 to 2,000 people, whereas the secondary units included households. Contrastingly, the primary sampling units in urban areas were blocks or groups of blocks of more than 2,000 individuals with an average of 140 households. Secondary sampling units were the same as in rural settings [17]. Details on the sampling process can be found in the technical documents of the ENDES [18].
Information on the dates of birth of all household members and the order in which these data were collected was used for the selection of participants. Only one participant aged 15 years or older was selected from each household. The participant with the closest birthday to the evaluation date was selected. In the event of a tie between two or more participants' birthdays (i.e. same birth day), the participant whose data was collected first was selected [19].
Participants of both sexes, who were of more than 15 years of age, living in both rural and urban areas and from every region in Peru, were included in ENDES. This study only included participants with complete data on the PHQ-9 and the sociodemographic variables of interest (sex, age, area, economic level, region, and year of evaluation).
The number of participants evaluated by the ENDES in each year is about 30,000 (see Table 1).

Variables
Depressive symptomatology in the last two weeks
Depressive symptomatology was evaluated using the Patient's Health Questionnaire (PHQ-9), available in the ENDES 2014-2018. The PHQ-9 was adapted for the population in Peru and had shown optimal values of reliability and validity when tested [20]. This tool has nine items based on the DSM-IV criteria and qualifies the response options from zero to three. PHQ-9 can, therefore, present a minimum score of 0 and a maximum score of 27. Depending on the total score, the PHQ-9 results may be: without indicators of depressive symptoms (score 0 to 4); mild depressive symptoms (score 5 to 9); moderate depressive symptoms (score 10 to 14); moderate-severe depressive symptoms (score 15 to 19); and severe depressive symptoms (score 20 and above) [21].
For this study, scores of ≥15 were used to define the presence of depressive symptoms, as this cut-off point presents better values of sensitivity (68%) and specificity (95%) [21]. In Peru, no studies have been conducted on the sensitivity and specificity of PHQ-9. Scores ≥15 were used to define the presence of depressive symptoms since it is an indicator of the presence of a major depressive episode that requires treatment [21,22].

For peer review only

Depressive symptomatology in the last year

A modified version of the PHQ-9 was used to assess depressive symptoms over the last year to help identify the occurrence of depressive symptoms over a more extended time. This version of the PHQ-9 was used in ENDES 2014-2018 to evaluate depressive symptoms experienced at some point in the past 12 months. Participants were asked to remember an event in the last 12 months in which they had discomfort or problems. Once the participants identified that troublesome or annoying event, the PHQ-9 was applied based on the two weeks around that event. The definition of depressive symptoms experienced some time in the last year was based on two criteria: 1) a score of ≥15 in the two-week version of PHQ-9, or 2) a score of ≥15 in the modified version of PHQ-9 for the last year. The age-standardized prevalence was also analyzed.

Proportion of depressive cases treated in the last year

A participant was considered to receive treatment for symptoms of depression based on two criteria:

1) Depressive symptoms had been recorded in the previous year or the past two weeks (PHQ-9 ≥ 15)
2) They had self-reported receiving treatment for depression from a health professional in the last 12 months.

There were three response options ("yes," "no," or "I do not remember"). Participants were only considered having received treatment if an affirmative answer was provided. It should be noted that ENDES data did not include information about the frequency or type of treatment received.

Other variables

In addition to the previous variables mentioned, a set of sociodemographic variables were also taken into consideration. The wealth level of participants was defined in quintiles (very low, low, middle, high, and very high) based on a wealth index available in the ENDES [23]. This index is calculated by using the availability of goods and services, the housing characteristics that the participants report having [17], and the quintiles of wealth. The index is performed within each cross-section (each year). The calculation of this index can be found in Rutstein and Johnson [23]. As a continuous variable, age was split into four groups (15 to 34, 35 to 54, 55 to 74, and 75 to more). The sex (male vs. female), the study area (urban/rural), and the year of ENDES evaluation were also taken into consideration.

Statistical methods

Main analysis

All statistical analyses were performed using Stata 13 (StataCorp, College Station, TX, USA). The graphics were elaborated using the ggplot libraries in R (version 3. 5. 1) and QGIS v2.18. All the analyses performed considered the design by a complex sampling of the ENDES, and the analyses performed were adjusted based on the weight factor provided by each ENDES assessment year. Adjustments were made with the STATA command "svy" for all analyses, except for the factor analysis (sub-analysis) where the "lavaan" and "lavaan.survey" package was used in R.

The number and proportion of participants excluded from analyses were recorded (see Supplement 1). A descriptive analysis of the participants was carried out for each year of ENDES. An analysis was conducted on subgroup participants reporting depressive symptoms over the past year in order to determine the proportion of those who reported receiving treatment.

The analysis was conducted at both country and regional levels. A 95% confidence interval (95% CI) was calculated for the prevalence at the regional and national levels. The trend over time was age-standardized and evaluated using the score test for trend; for this, the year 2014 was used as the reference category.

For peer review only - http://bmjopen.bmj.com/site/about/guidelines.xhtml

The age-standardized prevalence of depressive symptoms was estimated using the World Health Organization (WHO) population as the reference population [24]. The age-standardized prevalence is carried out since it allows us to compare our results with other studies carried out in other countries.

Sub-analysis

A sub-analysis was performed to compare sociodemographic characteristics in people receiving treatment for depressive symptoms in the last year. The Pearson χ^2 test was used to make these comparisons (see Supplement 3). Crude and adjusted models were created using a generalized linear model, assuming a Poisson distribution, link log, and robust variance was used as suggested in the literature [25]. Prevalence ratios (PR) and 95% CI were reported.

An additional post hoc analysis was performed, which conditions that participants with depressive symptoms must meet the DSM-5 criteria. This analysis aims to identify whether using the clinical criteria proposed by DSM-5 can alter the results (higher or lower) or be equivalent to our main results, which use a score of ≥15 in the PHQ-9. For this sub-analysis participants must have feelings of sadness or anhedonia (have a score of two or more on items 1 and 2 of the PHQ-9, i.e., “more than half the days” and “nearly every day”) and at least five of the other seven indicators (have a score of at least one on five of the other seven items of the PHQ-9).

To evaluate the measurement properties of the modified version of the PHQ-9 that collects information on depressive symptoms in the last year, a subanalysis was conducted (see Supplement 2). A confirmatory factor analysis was performed to evaluate the validity of the modified version of the PHQ-9, considering the ordinal nature of the items and using the estimate of weighted least squares means and variance adjusted [26]. It was considered that the instrument presents sufficient evidence of validity if it reaches optimum values in the different goodness-of-fit indices. The Comparative Fit Index (CFI) and the Tucker-Lewis Index (TLI) were used; these indices must be greater than 0.90 to be considered adequate levels [27]. The Root Mean Square Error of Approximation (RMSEA) with a confidence interval of 90% and the Standardized Root Mean Square Residual (SRMR) were also used, both indices considering fair values lower than 0.08 [27]. On the other hand, reliability was evaluated by the internal consistency coefficient of alpha and omega. Both coefficients consider that adequate levels of reliability are reached if they score higher than 0.80 [28].

Ethics

The data used in our study are openly accessible to the general public. They do not use any personal identifier (anonymous) and consequently do not represent an ethical risk for participants. The National Institute of Statistics and Informatics, a Peruvian government organization, was responsible for the collection of ENDES data. This institution requested the consent of participants to obtain the information required in the survey. For each person of legal age (18 years old and above), the informed consent for the collection of information was taken. In the case of minors (17 years old and younger), the request for consent was read to one of their parents or legal guardians to allow the evaluation of the minor.

Patient and Public Involvement

The patients or members of the public were not involved in the design, conduct, reporting, or dissemination plans of the research.

RESULTS

Participants

For peer review only

The ENDES between 2014 and 2018 included the data of 166,290 participants. The participation rate varied between 95.7% in 2014 and 97.4% in 2018. After excluding all records with incomplete information (n = 5,229, see Supplement 1), a total of 161,061 participants was included. Of these, 51.7% were women, the mean age was 40.3 years (SD = 17.1), and 73% lived in urban settings. The sociodemographic characteristics of the participants for each year are shown in Table 1.

The measurement properties of the modified version of the PHQ-9 to assess depressive symptoms during the last year were evaluated before the primary analyses. The modified version of the PHQ-9 was identified as having evidence of validity by confirmatory factor analysis (CFI=0.92; RMSEA=0.05) and evidence of reliability by internal consistency (ω=0.87; α=0.86). More information on the factorial analysis of the modified version of the PHQ-9 is presented in supplement 2.

Prevalence of depressive symptomatology

In Peru, between 2014 and 2018, the age-standardized prevalence of depressive symptoms in the last two weeks (score test for trend: p-value = 0.39, χ^2=0.72) and in the last year (score test for trend: p-value = 0.38, χ^2=0.74) did not show a significant trend. In 2018, the age-standardized prevalence in the last two weeks and in the last year was 2.3% (n=892, 95% CI: 2.1%−2.5%) and 6.2% (n=2,291, 95% CI: 5.8%−6.6%), respectively (See Figure 1A). On the other hand, in 2018, the un-standardized prevalence of depressive symptoms in the last two weeks was 2.7% (95% CI: 2.4%-2.9%) and in the last year was 6.2% (95% CI: 5.8%−6.6%) and therefore showed little to no difference from the age-standardized prevalence results.

Women had a higher age-standardized prevalence of depressive symptoms in the last two weeks and the last year, in comparison to men. Adults older than 75 years and individuals in the lowest wealth quintile were those with the highest age-standardized prevalence in the last two weeks and the last year (see Table 2). Figure 2 shows the age-standardized prevalence of depressive symptoms by region. There was no variation in the trends over time in any of the evaluated regions.

Trend results were not different when DSM-5 criteria were used. Thus, the age-standardized prevalence was very similar for both depressive symptoms in the last two weeks (3.4% in 2014 to 3.3% in 2018) and in the last year (6.8% in 2014 to 6.8% in 2018).

Proportion of depressive cases treated

At the national level, the proportion of depressive cases treated in the last year who reported receiving any treatment did not show any significant changes over time (score test for trend: p-value = 0.19, χ^2=1.66; see figure 1B). In 2018, 14.4% (n= 292, 95% CI: 12.2%−16.9%) of people with depressive symptoms in the last year reported having received some type of treatment from a healthcare professional. A higher proportion of women self-reporting for treatment was found when compared to men (only 2018, 15.9% in women and 11.1% in men). However, it was not found to be statistically significant (see Table 3).

The individuals in the highest wealth quintile (26.1% in 2018) have a much higher proportion of depressive cases treated than those in the lowest wealth quintile (6.2% in 2018). The probability of receiving treatment was almost five times greater in the highest wealth quintile compared to the lowest wealth quintile (RP=5.82, 95% IC: 2.54−10.18, see Table 3). No trend of sociodemographic characteristics between 2014 and 2018 was found. At the regional level, the highest proportion of depressive cases treated was on the coast (see figure 2C). The regions with the highest proportion of depressive cases treated were Callao (38.5%), and La Libertad (28.8%). Those with the lowest proportion of depressive cases treated were Puno (4.8%) and Huancavelica (5.5%), which are regions both in located the highlands (see Supplement 3).

For peer review only

When performing the subanalysis that includes the DSM-5 criteria, the trend results are not modified, and the probability of receiving treatment was very similar (14.5% in 2014 to 13.0% in 2018).

DISCUSSION

Main results

In Peru, from 2014 to 2018, no changes in prevalence rates (in the two weeks prior and over the last year) were found. Similarly, no change in the trends of treatment rates was found. The proportion of people with depressive symptoms receiving treatment was lower in people who live in rural areas and who are of a low level of wealth. Therefore, this situation could be generating a case of inequality in access to treatment in Peru, related to social determinants such as wealth and geographical location.

The results from this study, therefore, provide evidence for a need for an increased commitment and focus on the mental health reforms recently initiated by the Peruvian Ministry of Health. Despite strong evidence of a high prevalence of depression in the country, treatment rates remain low. Along with significant socioeconomic inequalities across the country, this requires an increased allocation of funds and additional resources to prevent and treat depression in the population.

Comparison with other studies

Prevalence of depressive symptomatology

When compared to other countries in Latin America, Peru has demonstrated a lower (un-standardized) prevalence of depressive symptoms than Brazil (9.7%) while sharing similar levels to those found in Colombia (5.1%) [29 30]. These findings have additionally been supported by other studies [1]. Contrastingly, high-income countries such as Australia (9.8%) and the United States (8.4%) have exhibited higher age-standardized prevalences than those found in Peru [31 32]. Other countries have reported that high-income countries, such as the USA and Australia, have a population with a higher proportion of people suffering from non-communicable and chronic diseases. There is evidence that people with chronic and non-communicable diseases have a higher proportion of depressive symptoms [33], hence the higher prevalence estimate in high-income countries. It should be noted that these studies may be overestimating the proportion of depressive symptoms. As the self-report instruments tend to have higher values than structured diagnostic interviews [34].

No variation in the prevalence of depressive symptoms was identified in Peru between 2014 and 2018. Our results are consistent with a meta-analysis of 116 epidemiological studies (1990 to 2010) [9] and with other epidemiological studies with only two measurements over several years conducted in Chile (2003 vs. 2010) and Germany (1997–1999 vs. 2009–2012) [35 36]. Contrastingly, three population-based studies found that there has been an increase in depressive symptoms. The first was carried out in the USA that measured prevalence over 11 years (2005 to 2015), the second was conducted for two years in Denmark (2000 vs. 2006), and the last was in northeast Germany between 1997-2001 and 2008-2012 [12 13 37]. The heterogeneity presented in these results can be explained by the variation in the number of measurements and the amount of time evaluated between the different studies. In order to obtain a stable trend, it is necessary to have consecutive measurements. Although our work only analyzed five-year trends, it is perhaps the first to report estimates across consecutive years using nationally representative surveys.

It should be noted that the studies, as mentioned earlier, evaluate only depressive symptoms and not major depressive disorders.

For peer review only

Proportion of depressive cases treated

The treatment gap reported by the Peruvian Ministry of Health (85.9% in 2017) is consistent with our results [38]. However, the treatment gap is higher than those reported in other countries such as the USA (72.4%) [39] or Chile (78.8%) [35]. This more massive gap could be explained by the stigma associated with seeking mental health care and treatment, preventing patients actually to seek professional attention [40]. Limited resources and a lack of trained health personnel could also play a role [38 41].

No significant variation was found by year in the proportion of depressive cases treated in Peru. This situation is different from that of USA [42] and the Republic of Georgia [43], where there has been a significant increase in the proportion of depressive cases treated. The difference could be attributed to the new mental health policies that these countries have implemented and the amount of money they invest in mental healthcare. Peru is undergoing a mental health reform focused on primary care with the implementation of Community Mental Health Centers (CMHCs)[16]. The effects at the population level, especially with regards to the proportion of the population with depressive symptoms receiving treatment has not yet been recorded. This could be because it is too early to observe the effects of the CMHCs, as this policy started in 2015 with 22 CMHCs, whereas by the end of 2018, there were 106 CMHCs throughout Peru. Besides, CMHCs are not distributed in a decentralized manner. In the region of Lima, there are only three specialized psychiatric hospitals in Peru and 20% of all CMHCs, and this could explain why the Callao region (with only 147 km^2 of surface and located very close to Lima) has the highest proportion of depressive cases treated (23.8%). This region, despite its small size, has 3 CMHCs. It is very close to the three psychiatric hospitals in Peru and other CMHCs. In comparison with Puno, which has the lowest proportion of depressive cases treated (4.8%), possibly since it has only 2 CMHCs to supply the territory of 66,997 km^2. Although largely speculative, these data suggest that the treatment gap could be reduced if CMHCs and other mental health services were available in other regions.

Prevalence and treatment of depressive symptomatology

It could be argued that increasing the proportion of people receiving treatment can help reduce the prevalence of depressive symptoms, but this does not seem supported by evidence. A study conducted in four high-income countries identified that a higher proportion of care is not related to reducing the prevalence of mental health problems [44]. On the other hand, a review suggests that the prevalence of depressive symptoms is reduced if the quality of mental health care is improved, waitlists are eradicated, antidepressants are prescribed with greater caution, or psychological treatments are opted for, and preventive interventions linked to the community are carried out [45].

Relevance in public health

The prevalence of depressive symptoms in the last year in Peru was similar to other countries; however, only 14.1% of first-level care centers provide mental health services [38]. This situation represents a public health concern as the lack of access to treatment is considered one of the causes of the treatment gap of mental health experienced by the Peruvian health system [41]. The Peruvian mental health reform seeks, among other objectives, to improve access to mental health care. Nonetheless, our results indicate that it is still too early to see the effects of this reform. Since the treatment gap has not been reduced at the population level in recent years (between 2014 to 2018), at least not in people with depressive symptoms. Despite this, the implementation of CMHCs seems to be a beneficial policy since the health networks that implemented a CMHC increased up to four times the number of mental health care consultations, compared to before its implementation [38]. Based on this, we could expect that the implementation of new CMHCs might

increase the number of those suffering from mental health problems being attended to, and thereby reduce the treatment gap.

There is evidence that people with a diagnosis of depression are more likely to receive treatment [46], and the implementation of screening for mental health problems in primary care, this has proven to be very useful in other countries to improve the diagnosis and follow-up [47 48]. However, population-based screening for depressive symptoms –or other mental health problems– has not been implemented in any Peruvian clinical guidelines [49 50]. Even though CMHCs aim to screen for mental health problems, their capacity is still limited; thus, it is not feasible to cover large populations. Even if other primary care facilities conducted this screening initiative, there is a limited number of psychologists, psychiatrists, and primary care physicians who can perform these screening tests [41]. Therefore, there should be a population-based screening plan be implemented; it would be necessary to involve lay people (e.g., community health workers) to fill the gap in trained health personnel. Finally, for a clinical practice guideline to be successfully implemented and accepted by the patients, they should be involved in the development of these guidelines [51].

Strengths and limitations

The present study includes the analysis of five measurements from different years of information from nationally-representative surveys of the Peruvian population . This is the first study assessing the trends of depressive symptoms in Peru. However, this study has limitations. First, the evaluation period may have been short (between 2014 to 2018). If prevalence or treatment were to increase, it most likely is small but sustained annually. So it would be necessary to have a higher number of measurements (10 or 20 years) in order to be able to identify a trend of higher or a decrease of depression symptoms. Second, although the evaluation of depressive symptoms was performed using a valid tool [52], this does not replace an evaluation conducted by a psychiatrist; thus, misclassification may be an issue. Third, another element that could generate bias is the cut-off point used to classify people with depressive symptoms (PHQ-9 scores ≥15). However, when the post hoc analysis was performed using the DSM-5 criteria, the main results were identified as the same (no change in prevalence trends and no proportion of depressive cases treated). Finally, other important variables that would have allowed a better understanding of the results were not included. For example, type of treatment received, duration of treatment, and if the participant has previously received a diagnosis of depression.

Conclusions

No significant trend was found in the age-standardized prevalence of depressive symptoms (in the last two weeks and the last year) or the proportion of depressive cases treated. The proportion of depressive cases treated was lower in people with depressive symptoms who lived in rural areas and who were of a low level of wealth. It is necessary to implement policies and interventions to reduce the treatment gap and the prevalence of depressive symptoms accounting for social inequalities across the country.

CONTRIBUTORSHIP STATEMENT

David Villarreal-Zegarra: Conceived and designed the analysis; collected the data; contributed data or analysis tools; performed the analysis; wrote the paper.

Milagros Cabrera-Alva: Contributed data or analysis tools; wrote the paper.

Rodrigo M Carrillo-Larco: Conceived and designed the analysis; contributed data or analysis tools; performed the analysis; wrote the paper; supervision of the paper.

For peer review only

Antonio Bernabe-Ortiz: Conceived and designed the analysis; contributed data or analysis tools; performed the analysis; wrote the paper; supervision of the paper.

COMPETING INTERESTS

The authors have declared that no competing interests exist.

FUNDING

This work was supported by the Strategic Award, Wellcome Trust-Imperial College Centre for Global Health Research (100693/Z/12/Z), Imperial College London Wellcome Trust Institutional Strategic Support Fund [Global Health Clinical Research Training Fellowship] (294834/Z/16/Z ISSF ICL), and Rodrigo M Carrillo-Larco are supported by a Wellcome Trust International Training Fellowship (214185/Z/18/Z). The funders had no role in study design, data collection, and analysis, decision to publish, or preparation of the manuscript.

GRANT DISCLOSURES

The authors disclosed the following grant information:

Strategic Award, Wellcome Trust-Imperial College Centre for Global Health Research: 100693/Z/12/Z.

Imperial College London Wellcome Trust Institutional Strategic Support Fund: 294834/Z/16/Z ISSF ICL.

Wellcome Trust International Training Fellowship: 214185/Z/18/Z.

DATA SHARING STATEMENT

The database is freely accessible from the website of the National Institute of Statistics of Peru, URL: http://iinei.inei.gob.pe/microdatos/

The information can be obtained by entering the survey query tab and selecting the ENDES data using the health module data. Only cross-sectional information from 2014 to 2018 for the ENDES Health Questionnaire was used.

ACKNOWLEDGMENTS

DVZ thanks to Helena Kesar and Qory Moscoso for their style review in the final version of the English manuscript.

ABBREVIATIONS

CMHCs: Community Mental Health Centers.
DSM-IV: Diagnostic and Statistical Manual of Mental Disorders, 4th Edition.
DSM-5: Diagnostic and Statistical Manual of Mental Disorders, 5th Edition.
ENDES: Demographic and Health Survey of Peru.
SD: Standard Deviation.
PHQ-9: Patient's Health Questionnaire.
PR: Prevalence Ratios.
USA: United States of America.
WHO: World Health Organization.
95%CI: 95% confidence interval.

REFERENCES

For peer review only

1. World Health Organization. Depression and Other Common Mental Disorders. Global Health Estimates. Geneve: World Health Organization 2017.
2. Hsieh CR, Qin X. Depression hurts, depression costs: The medical spending attributable to depression and depressive symptoms in China. *Health economics* 2018;27(3):525-44. doi: 10.1002/hec.3604 [published Online First: 2017/10/11]
3. Vasiliadis HM, Dezetter A, Latimer E, et al. Assessing the Costs and Benefits of Insuring Psychological Services as Part of Medicare for Depression in Canada. *Psychiatric services* 2017;68(9):899-906. doi: 10.1176/appi.ps.201600395 [published Online First: 2017/05/16]
4. World Health Organization. The global burden of disease: 2004 update. Geneva: WHO 2008.
5. Salk RH, Hyde JS, Abramson LY. Gender differences in depression in representative national samples: Meta-analyses of diagnoses and symptoms. *Psychol Bull* 2017;143(8):783-822. doi: 10.1037/bul0000102 [published Online First: 2017/04/28]
6. Shao J, Li D, Zhang D, et al. Birth cohort changes in the depressive symptoms of Chinese older adults: a cross-temporal meta-analysis. *International Journal of Geriatric Psychiatry* 2013;28(11):1101-08. doi: doi:10.1002/gps.3942
7. Lee E-W, Park J-H. Individual and Socioeconomic Contextual Effects on Depressive Symptom in Korea: Multilevel Analysis of Cross-sectional Nationwide Survey. *Journal of Korean medical science* 2015;30(2):186-93.
8. Eaton J, McCay L, Semrau M, et al. Scale up of services for mental health in low-income and middle-income countries. *Lancet (London, England)* 2011;378(9802):1592-603. doi: 10.1016/s0140-6736(11)60891-x [published Online First: 2011/10/20]
9. Baxter AJ, Scott KM, Ferrari AJ, et al. Challenging the myth of an "epidemic" of common mental disorders: trends in the global prevalence of anxiety and depression between 1990 and 2010. *Depression and Anxiety* 2014;31(6):506-16. doi: doi:10.1002/da.22230
10. GBD 2016 Disease and Injury Incidence and Prevalence Collaborators. Global, regional, and national incidence, prevalence, and years lived with disability for 328 diseases and injuries for 195 countries, 1990-2016: a systematic analysis for the Global Burden of Disease Study 2016. *Lancet (London, England)* 2017;390(10100):1211-59. doi: 10.1016/s0140-6736(17)32154-2 [published Online First: 2017/09/19]
11. GBD 2015 Disease and Injury Incidence and Prevalence Collaborators. Global, regional, and national incidence, prevalence, and years lived with disability for 310 diseases and injuries, 1990-2015: a systematic analysis for the Global Burden of Disease Study 2015. *The Lancet* 2016;388(10053):1545-602. doi: 10.1016/s0140-6736(16)31678-6 [published Online First: 2016/10/14]
12. Andersen I, Thielen K, Bech P, et al. Increasing prevalence of depression from 2000 to 2006. *Scandinavian Journal of Public Health* 2011;39(8):857-63. doi: 10.1177/1403494811424611
13. Weinberger AH, Gbedemah M, Martinez AM, et al. Trends in depression prevalence in the USA from 2005 to 2015: widening disparities in vulnerable groups. *Psychological medicine* 2018;48(8):1308-15. doi: 10.1017/s0033291717002781 [published Online First: 2017/10/13]
14. Guerra M, Prina AM, Ferri CP, et al. A comparative cross-cultural study of the prevalence of late life depression in low and middle income countries. *Journal of Affective Disorders* 2016;190:362-68. doi: https://doi.org/10.1016/j.jad.2015.09.004
15. Thornicroft G, Chatterji S, Evans-Lacko S, et al. Undertreatment of people with major depressive disorder in 21 countries. *British Journal of Psychiatry* 2018;210(2):119-24. doi: 10.1192/bjp.bp.116.188078 [published Online First: 01/02]

For peer review only

16. Toyama M, Castillo H, Galea JT, et al. Peruvian Mental Health Reform: A Framework for Scaling-up Mental Health Services. *International journal of health policy and management* 2017;6(9):501-08. doi: 10.15171/ijhpm.2017.07 [published Online First: 2017/09/28]
17. Instituto Nacional de Estadística e Informática. Perú: encuesta demográfica y de salud familiar ENDES 2014. Perú: Instituto Nacional de Estadística e Informática 2015.
18. Instituto Nacional de Estadística e Informática. Perú - Encuesta Demográfica y de Salud Familiar ENDES 2017. Peru: Instituto Nacional de Estadística e Informática 2018.
19. Instituto Nacional de Estadística e Informática. Manual de la entrevistadora - Encuesta demográfica y de salud familiar. Perú: Instituto Nacional de Estadística e Informática 2018.
20. Villarreal-Zegarra D, Copez-Lonzoy A, Bernabe-Ortiz A, et al. Valid group comparisons can be made with the Patient Health Questionnaire (PHQ-9): A measurement invariance study across groups by demographic characteristics. *PloS one* 2019;14(9):e0221717. doi: 10.1371/journal.pone.0221717 [published Online First: 2019/09/10]
21. Kroenke K, Spitzer RL, Williams JB. The PHQ-9: validity of a brief depression severity measure. *J Gen Intern Med* 2001;16(9):606-13. [published Online First: 2001/09/15]
22. Manea L, Gilbody S, McMillan D. A diagnostic meta-analysis of the Patient Health Questionnaire-9 (PHQ-9) algorithm scoring method as a screen for depression. *General hospital psychiatry* 2015;37(1):67-75. doi: 10.1016/j.genhosppsych.2014.09.009 [published Online First: 2014/12/03]
23. Rutstein SO, Johnson K. The DHS wealth index. DHS comparative reports no. 6. Calverton, Maryland: ORC Macro 2004.
24. Ahmad OB, Boschi-Pinto C, Lopez AD, et al. Age standardization of rates: a new who standard. World Health Organization: Geneva 2001.
25. Barros AJ, Hirakata VN. Alternatives for logistic regression in cross-sectional studies: an empirical comparison of models that directly estimate the prevalence ratio. *BMC Med Res Methodol* 2003;3(1):21.
26. Brown TA. Confirmatory factor analysis for applied research. Second ed. New York: The Guilford Press 2015.
27. Hair JFA, R.E.; Tatham, R.L. & Black, W. Análisis Multivariante. Madrid: Prentice Hall 1999.
28. Ten Berge JM, Sočan G. The greatest lower bound to the reliability of a test and the hypothesis of unidimensionality. *Psychometrika* 2004;69(4):613-25.
29. Gómez-Restrepo C, Tamayo Martínez N, Bohórquez A, et al. Trastornos depresivos y de ansiedad y factores asociados en la población adulta colombiana, Encuesta Nacional de Salud Mental 2015. *Revista Colombiana de Psiquiatría* 2016;45:58-67. doi: https://doi.org/10.1016/j.rcp.2016.04.009
30. Barros MBA, Lima MG, Azevedo RCS, et al. Depression and health behaviors in Brazilian adults - PNS 2013. *Rev Saude Publica* 2017;51(suppl 1):8s. doi: 10.1590/s1518-8787.2017051000084 [published Online First: 2017/06/08]
31. Zhao G, Ford ES, Dhingra S, et al. Depression and anxiety among US adults: associations with body mass index. *International journal of obesity (2005)* 2009;33(2):257-66. doi: 10.1038/ijo.2008.268 [published Online First: 2009/01/07]
32. Atlantis E, Goldney RD, Eckert KA, et al. Trends in health-related quality of life and health service use associated with comorbid diabetes and major depression in South Australia, 1998-2008. *Social psychiatry and psychiatric epidemiology* 2012;47(6):871-7. doi: 10.1007/s00127-011-0394-4 [published Online First: 2011/05/19]
33. DeJean D, Giacomini M, Vanstone M, et al. Patient experiences of depression and anxiety with chronic disease: a systematic review and qualitative meta-synthesis. *Ontario health technology assessment series* 2013;13(16):1-33. [published Online First: 2013/11/15]

For peer review only

34. Lowe B, Kroenke K, Herzog W, et al. Measuring depression outcome with a brief self-report instrument: sensitivity to change of the Patient Health Questionnaire (PHQ-9). *J Affect Disord* 2004;81(1):61-6. doi: 10.1016/s0165-0327(03)00198-8 [published Online First: 2004/06/09]
35. Markkula N, Zitko P, Pena S, et al. Prevalence, trends, correlates and treatment of depression in Chile in 2003 to 2010. *Social psychiatry and psychiatric epidemiology* 2017;52(4):399-409. doi: 10.1007/s00127-017-1346-4 [published Online First: 2017/02/16]
36. Bretschneider J, Janitza S, Jacobi F, et al. Time trends in depression prevalence and health-related correlates: results from population-based surveys in Germany 1997–1999 vs. 2009–2012. *BMC psychiatry* 2018;18(1):394. doi: 10.1186/s12888-018-1973-7
37. Baumeister SE, Schomerus G, Schmidt CO, et al. Change in depressive symptoms and mental health-related quality of life in northeast Germany between 1997-2001 and 2008-2012. *International journal of public health* 2015;60(1):33-9. doi: 10.1007/s00038-014-0632-0 [published Online First: 2014/12/17]
38. Ministerio de Salud. Plan Nacional de Fortalecimiento de Servicios de Salud Mental Comunitaria 2018 – 2021. Perú: Ministerio de Salud 2018.
39. Wittayanukorn S, Qian J, Hansen RA. Prevalence of depressive symptoms and predictors of treatment among U.S. adults from 2005 to 2010. *Gen Hosp Psychiatry* 2014;36(3):330-6. doi: 10.1016/j.genhosppsych.2013.12.009 [published Online First: 2014/01/28]
40. Pattyn E, Verhaeghe M, Sercu C, et al. Public stigma and self-stigma: differential association with attitudes toward formal and informal help seeking. *Psychiatric services (Washington, DC)* 2014;65(2):232-8. doi: 10.1176/appi.ps.201200561 [published Online First: 2013/11/16]
41. Grupo del Banco Mundial. Movimiento por el cuidado comunitario de la salud mental en el Perú. Lima: Grupo del Banco Mundial 2019.
42. Mojtabai R, Olfson M, Han B. National Trends in the Prevalence and Treatment of Depression in Adolescents and Young Adults. *Pediatrics* 2016;138(6) doi: 10.1542/peds.2016-1878 [published Online First: 2016/12/13]
43. Kenchadze V, Chkonia E, Beria Z. Trends in the use of outpatient psychiatric services in the republic of Georgia. *Georgian medical news* 2013(215):60-5. [published Online First: 2013/03/14]
44. Jorm AF, Patten SB, Brugha TS, et al. Has increased provision of treatment reduced the prevalence of common mental disorders? Review of the evidence from four countries. *World psychiatry : official journal of the World Psychiatric Association (WPA)* 2017;16(1):90-99. doi: 10.1002/wps.20388 [published Online First: 2017/01/28]
45. Ormel J, Kessler RC, Schoevers R. Depression: more treatment but no drop in prevalence: how effective is treatment? And can we do better? *Current opinion in psychiatry* 2019;32(4):348-54. doi: 10.1097/yco.0000000000000505 [published Online First: 2019/03/12]
46. Fujii RK, Goren A, Annunziata K, et al. Prevalence, Awareness, Treatment, and Burden of Major Depressive Disorder: Estimates from the National Health and Wellness Survey in Brazil. *Value in health regional issues* 2012;1(2):235-43. doi: 10.1016/j.vhri.2012.09.011 [published Online First: 2012/12/01]
47. Siu AL, Bibbins-Domingo K, Grossman DC, et al. Screening for Depression in Adults: US Preventive Services Task Force Recommendation Statement. *Jama* 2016;315(4):380-7. doi: 10.1001/jama.2015.18392 [published Online First: 2016/01/28]
48. Vilagut G, Forero CG, Barbaglia G, et al. Screening for Depression in the General Population with the Center for Epidemiologic Studies Depression (CES-D): A Systematic Review with

For peer review only

Meta-Analysis. *PloS one* 2016;11(5):e0155431. doi: 10.1371/journal.pone.0155431 [published Online First: 2016/05/18]

49. Ministerio de Salud. Guía de practica clínica en depresión. Perú: Ministerio de Salud 2008.
50. Instituto Nacional de Salud. Guía de práctica clínica para el tratamiento de depresión en adultos - versión extensa. Lima: Instituto Nacional de Salud 2017.
51. Zafra-Tanaka JH, Goicochea-Lugo S, Villarreal-Zegarra D, et al. Characteristics and quality of clinical practice guidelines for depression in adults: a scoping review. *BMC psychiatry* 2019;19(1):76. doi: 10.1186/s12888-019-2057-z
52. Baader M T, Molina F JL, Venezian B S, et al. Validación y utilidad de la encuesta PHQ-9 (Patient Health Questionnaire) en el diagnóstico de depresión en pacientes usuarios de atención primaria en Chile. *Rev Chil Neuropsiquiatr* 2012;50:10-22.

For peer review only

FIGURES AND TABLES

Figure 1. Age-standardized prevalence of depressive symptoms and proportion of depressive cases treated in Peru between 2014 to 2018.
A) Age-standardized prevalence of depressive symptoms in the last year and the last two weeks in Peru by year. B) Proportion of depressive cases treated in the last year in Peru by year. All the analyzes, the weighted proportion by complex sampling was used.

Figure 2. Age-standardized prevalence of depressive symptoms and proportion of depressive cases treated in Peru by region in 2018.
A) Age-standardized prevalence of depressive symptoms in the last two weeks from Peru for a region in 2018. B) Age-standardized prevalence of depressive symptoms in the last year in Peru for a region in 2018. C) Proportion of depressive cases treated in the last year for the region in 2018. Two-stage sample design was taken into account for percentage estimations.
Figure designed by the authors.

For peer review only

Table 1. Descriptive characteristics of the participants included in the study by year.

		2014 (n=27,633)		2015 (n=33,573)		2016 (n=32,373)		2017 (n=33,794)		2018 (n=34,476)	
		n	%	*n*	%	*n*	%	*n*	%	*n*	%
Sex	Male	12,806	46.3%	14,788	48.9%	14,126	48.9%	14,424	48.5%	14,696	48.4%
	Female	14,827	53.7%	18,573	51.1%	18,247	51.1%	18,794	51.5%	19,780	51.6%
Age	15-34	11,022	43.1%	16,576	43.1%	15,498	44.4%	15,913	43.6%	16,121	42.7%
	35-54	9,395	33.9%	10,887	33.9%	10,740	33.8%	10,819	34.5%	11,692	34.6%
	55-74	5,460	18.0%	4,685	18.0%	4,791	16.8%	5,030	17.0%	5,332	17.7%
	75+	1,756	5.0%	1,213	5.0%	1,344	5.0%	1,456	4.9%	1,331	5.0%
Area	Rural	10,663	24.9%	11,453	34.5%	11,113	35.2%	11,349	20.8%	11,923	19.6%
	Urban	16,970	75.1%	21,908	65.5%	21,260	64.8%	21,869	79.3%	22,553	80.4%
Wealth index	Very low	8,151	18.7%	9,370	26.3%	8,967	26.4%	10,045	18.5%	11,019	18.7%
	Low	6,782	19.2%	8,376	21.2%	8,464	22.0%	8,575	20.8%	8,514	20.6%
	Middle	5,153	19.9%	6,459	18.3%	6,361	18.5%	6,368	21.0%	6,379	20.9%
	High	4,103	21.0%	5,089	17.7%	5,056	17.4%	4,846	20.3%	4,909	20.4%
	Very high	3,444	21.2%	4,067	16.4%	3,525	15.8%	3,384	19.4%	3,655	19.4%

Note: Two-stage sample design was taken into account for percentage estimations.

For peer review only

Table 2. Age-standardized prevalence of depressive symptoms and proportion of depressive cases treated in the last year in Peru by selected socio-demographic characteristics (2014-2018, with cut-off point 15).

			2014	2015	2016	2017	2018
Age-standardized prevalence of Depressive symptoms in the last two week †	Sex	Male	1.5%	1.9%	1.9%	1.3%	1.1%
		Female	3.6%	3.9%	4.0%	3.3%	3.4%
	Age	15-34	1.5%	1.5%	1.6%	1.2%	1.1%
		35-54	2.9%	2.6%	2.6%	2.5%	2.3%
		55-74	4.0%	5.7%	5.0%	4.1%	4.1%
		75+	6.7%	9.0%	11.2%	6.0%	6.5%
	Area	Rural	2.9%	3.5%	3.4%	3.2%	3.3%
		Urban	2.5%	2.5%	2.7%	2.1%	2.0%
	Wealth index	Very low	3.0%	3.8%	3.6%	3.2%	3.5%
		Low	3.1%	3.3%	2.8%	3.1%	2.7%
		Middle	3.3%	3.2%	3.5%	2.8%	2.4%
		High	2.5%	2.1%	2.6%	1.6%	1.7%
		Very high	1.6%	1.7%	1.7%	1.2%	1.2%
Age-standardized prevalence of Depressive symptoms in the last year †	Sex	Male	3.7%	4.3%	4.0%	3.5%	3.9%
		Female	8.5%	9.1%	9.3%	8.2%	8.2%
	Age	15-34	4.5%	4.4%	4.6%	4.1%	4.3%
		35-54	6.9%	6.7%	6.5%	6.0%	6.1%
		55-74	8.1%	10.6%	9.7%	9.3%	9.0%
		75+	12.5%	15.3%	17.3%	9.6%	13.2%
	Area	Rural	7.2%	7.9%	7.6%	7.4%	8.1%
		Urban	5.9%	6.0%	6.1%	5.5%	5.6%
	Wealth index	Very low	7.4%	8.1%	7.8%	7.3%	8.4%
		Low	7.4%	7.7%	7.1%	7.4%	7.1%
		Middle	7.1%	6.9%	7.4%	6.5%	6.6%
		High	5.4%	5.7%	5.9%	4.6%	5.0%
		Very high	4.8%	4.5%	4.3%	4.3%	4.3%
Proportion of depressive cases treated in the last year ‡	Sex	Male	13.3%	11.9%	10.7%	10.6%	11.1%
		Female	15.1%	14.7%	16.9%	16.6%	15.9%
	Age	15-34	15.7%	14.8%	19.7%	19.2%	14.7%
		35-54	17.3%	16.1%	18.0%	15.9%	13.6%
		55-74	12.1%	12.2%	10.5%	10.0%	16.0%
		75+	7.4%	8.5%	6.2%	10.0%	12.5%
	Area	Rural	7.9%	7.2%	7.1%	5.6%	7.8%
		Urban	17.5%	18.6%	21.0%	18.4%	16.9%
	Wealth index	Very low	5.8%	5.7%	5.8%	3.3%	6.2%
		Low	8.8%	11.6%	14.1%	13.2%	10.1%
		Middle	16.3%	13.8%	17.1%	15.3%	17.4%
		High	20.3%	22.0%	24.7%	14.2%	20.4%
		Very high	27.1%	31.3%	31.0%	38.4%	26.1%

Note: Two-stage sample design was taken into account for percentage estimations. † The analysis considered the total of the Peruvian population. ‡ An analysis is done by subgroups, considering only people who have depressive symptoms.

For peer review only

Table 3. Association between receiving treatment and sociodemographic characteristics in people with depressive symptoms in last year, only in 2018.

		Model crude PR (95% CI)	Adjusted model * PR (95% CI)
Sex	Male	1	1
	Female	1.42 (0.96 - 2.10)	1.30 (0.88 - 1.92)
Age	15-34	1	1
	35-54	0.92 (0.64 - 1.32)	0.98 (0.69 - 1.41)
	55-74	1.09 (0.71 - 1.67)	1.23 (0.81 - 1.87)
	75+	0.85 (0.37 - 1.91)	1.08 (0.49 - 2.39)
Area	Rural	1	1
	Urban	**2.18 (1.58 - 3.00)**	0.81 (0.48 - 1.37)
Wealth index	Very low	1	1
	Low	**1.65 (1.05 - 2.60)**	**1.89 (1.06 - 3.38)**
	Middle	**2.84 (1.78 - 4.50)**	**3.36 (1.73 - 6.53)**
	High	**3.32 (2.04 - 5.40)**	**3.92 (1.96 - 7.87)**
	Very high	**4.25 (2.65 - 6.81)**	**5.08 (2.54 - 10.18)**

Note: Analysis is done by subgroups, considering only people who have depressive symptoms, the complex sampling was used.
* Adjusted by sex, age, wealth index, and area.

For peer review only

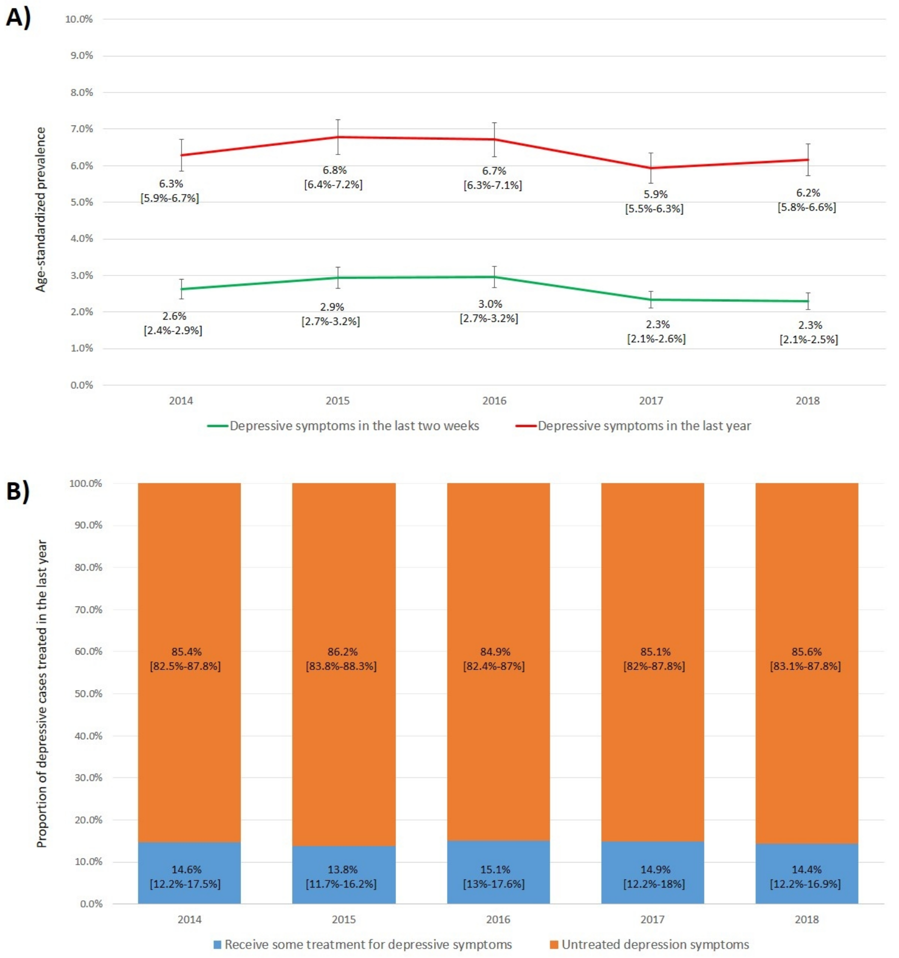

Figure 1. Age-standardized prevalence of depressive symptoms and proportion of depressive cases treated in Peru between 2014 to 2018.

350x350mm (96 x 96 DPI)

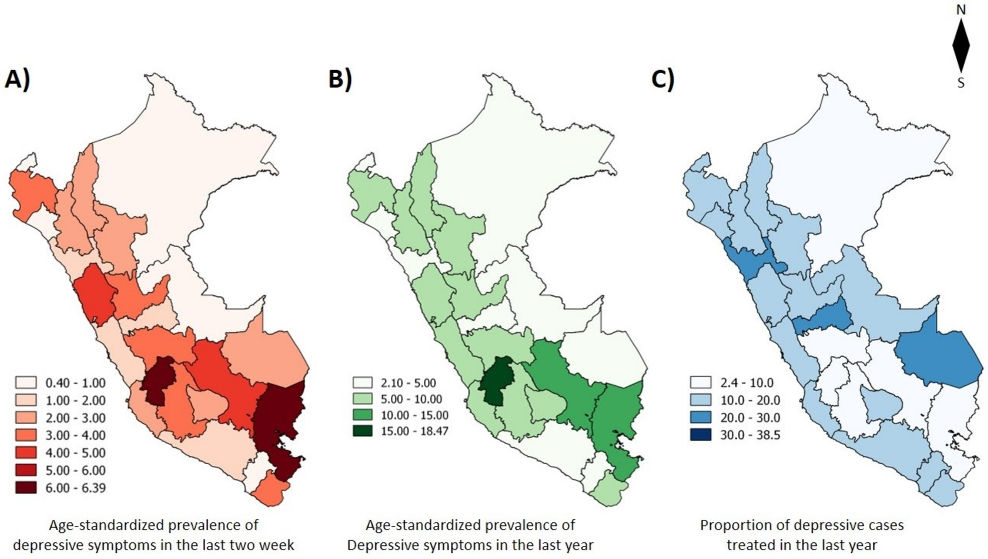

Figure 2. Age-standardized prevalence of depressive symptoms and proportion of depressive cases treated in Peru by region in 2018.

343x200mm (96 x 96 DPI)

Supplement 1. Descriptive characteristics of the participants not included in the study (2014-2018).

		2014		2015		2016		2017		2018	
		n	%	*n*	%	*n*	%	*n*	%	*n*	%
Sex	Male	0	0.0%	1	0.1%	0	0.0%	0	0.0%	0	0.00%
	Female	0	0.0%	8	0.7%	0	0.0%	1	0.1%	0	0.00%
	Missing	1,160	100.0%	1,101	99.2%	1,166	100.0%	880	99.9%	912	100.00%
Age	15-34	0	0.0%	4	0.3%	0	0.0%	0	0.0%	0	0.00%
	35-54	0	0.0%	3	0.3%	0	0.0%	0	0.0%	0	0.00%
	55-74	0	0.0%	1	0.1%	0	0.0%	0	0.0%	0	0.00%
	75+	0	0.0%	1	0.1%	0	0.0%	74	0.1%	0	0.00%
	Missing	1,160	100.0%	1,101	99.2%	1,166	100.0%	880	99.9%	912	100.00%
Area	Rural	261	22.5%	289	26.0%	253	21.7%	194	22.0%	175	19.2%
	Urban	899	77.5%	821	74.0%	913	78.3%	687	78.0%	737	80.8%
	Missing	0	0.0%	0	0.0%	0	0.0%	0	0.0%	0	0.00%
Wealth index	Very low	207	17.8%	261	23.5%	214	18.4%	181	20.5%	155	17.0%
	Low	205	17.7%	201	18.1%	209	17.9%	152	17.3%	162	17.8%
	Middle	230	19.8%	192	17.3%	232	19.9%	152	17.3%	199	21.8%
	High	241	20.8%	225	20.3%	258	22.1%	204	23.2%	159	17.4%
	Very high	277	23.9%	231	20.8%	253	21.7%	192	21.8%	237	26.0%
	Missing	0	0.0%	0	0.0%	0	0.0%	0	0.0%	0	0.00%

For peer review only

Supplement 2. Factor loads, goodness-of-fit indices, and internal consistency coefficients of the modified version of the PHQ-9 to evaluate depressive symptoms in the past year, using 2018 data.

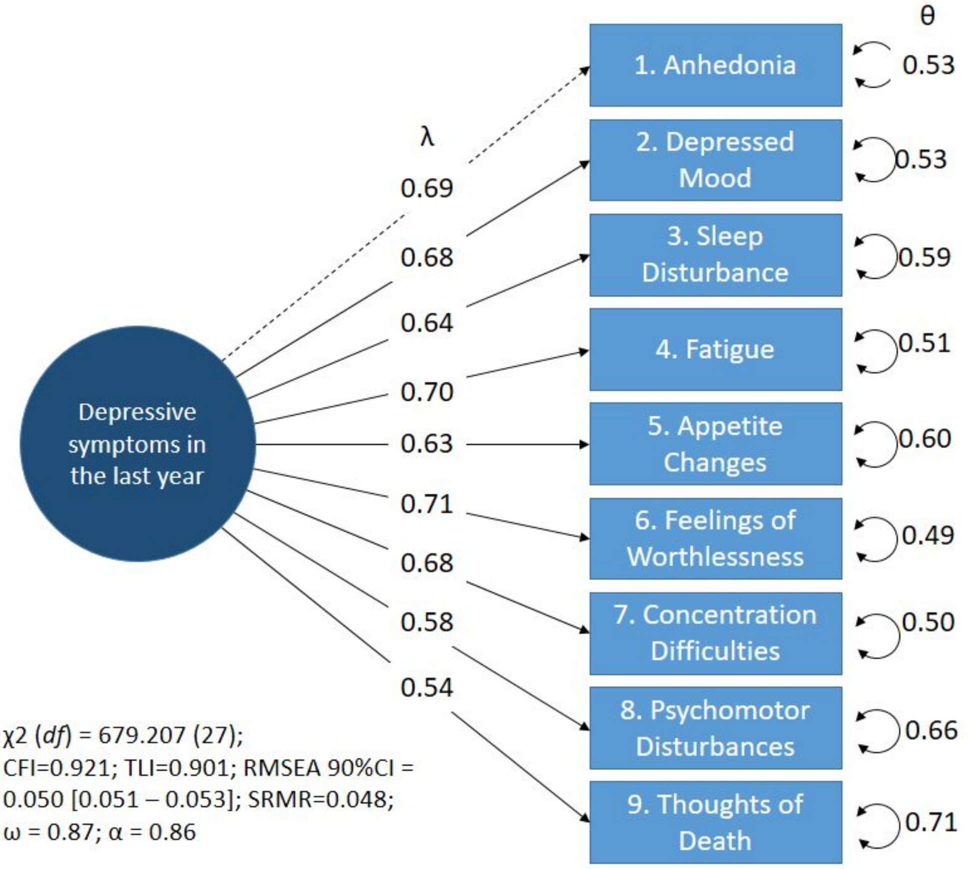

Supplement 3. Age-standardized prevalence of depressive symptoms and proportion of treatment in Peru by region in 2018 (with cut-off point 15).

Region	Age-standardized prevalence of Depressive symptoms in the last two week *	Age-standardized prevalence of Depressive symptoms in the last year *	Proportion of People with depressive symptoms receiving treatment **
Amazonas	2.19%	5.63%	11.77%
Ancash	4.03%	9.95%	10.67%
Apurímac	2.52%	7.81%	16.63%
Arequipa	1.20%	4.19%	19.51%
Ayacucho	3.68%	8.93%	8.27%
Cajamarca	2.47%	5.54%	11.48%
Callao	1.70%	4.51%	38.51%
Cusco	4.36%	12.36%	9.74%
Huancavelica	6.10%	18.47%	5.89%
Huánuco	3.62%	7.95%	17.87%
Ica	2.99%	5.78%	15.42%
Junín	3.12%	6.87%	9.79%
La Libertad	1.41%	4.15%	28.52%
Lambayeque	0.40%	2.10%	17.19%
Lima	1.69%	5.40%	15.77%
Loreto	0.80%	2.20%	2.38%
Madre de Dios	2.12%	3.63%	22.03%
Moquegua	0.84%	3.36%	10.07%
Pasco	1.27%	4.61%	24.52%
Piura	3.21%	7.91%	14.08%
Puno	6.39%	13.71%	7.12%
San Martín	2.05%	5.16%	12.92%
Tacna	3.86%	7.21%	12.34%
Tumbes	0.88%	2.52%	13.03%
Ucayali	0.75%	3.39%	14.51%

Note: Two-stage sample design was taken into account for percentage estimations. * The analysis considered the total of Peruvian population. ** An analysis is done by subgroups, considering only people who have depressive symptoms.

For peer review only

STROBE Statement—Checklist of items that should be included in reports of ***cross-sectional studies***

	Item No	Recommendation	Page No
**Title and abstract**	1	(*a*) Indicate the study's design with a commonly used term in the title or the abstract	pp.1
		(*b*) Provide in the abstract an informative and balanced summary of what was done and what was found	pp.2
**Introduction**			
Background/rationale	2	Explain the scientific background and rationale for the investigation being reported	pp.4
Objectives	3	State specific objectives, including any prespecified hypotheses	pp.4 paragraph 5
**Methods**			
Study design	4	Present key elements of study design early in the paper	pp.4
Setting	5	Describe the setting, locations, and relevant dates, including periods of recruitment, exposure, follow-up, and data collection	pp.4 paragraph 6
Participants	6	(*a*) Give the eligibility criteria, and the sources and methods of selection of participants	pp.5 paragraph 3
Variables	7	Clearly define all outcomes, exposures, predictors, potential confounders, and effect modifiers. Give diagnostic criteria, if applicable	pp.5 paragraph 8
Data sources/ measurement	8*	For each variable of interest, give sources of data and details of methods of assessment (measurement). Describe comparability of assessment methods if there is more than one group	pp.5 paragraph 8
Bias	9	Describe any efforts to address potential sources of bias	pp.5 paragraph 8
Study size	10	Explain how the study size was arrived at	NA
Quantitative variables	11	Explain how quantitative variables were handled in the analyses. If applicable, describe which groupings were chosen and why	pp.5 paragraph 8
Statistical methods	12	(*a*) Describe all statistical methods, including those used to control for confounding	pp.6
		(*b*) Describe any methods used to examine subgroups and interactions	pp.6
		(*c*) Explain how missing data were addressed	pp.7
		(*d*) If applicable, describe analytical methods taking account of sampling strategy	pp.7
		(*e*) Describe any sensitivity analyses	pp.7
**Results**			
Participants	13*	(a) Report numbers of individuals at each stage of study—eg numbers potentially eligible, examined for eligibility, confirmed eligible, included in the study, completing follow-up, and analysed	pp.8 paragraph 1
		(b) Give reasons for non-participation at each stage	pp.8 paragraph 2
		(c) Consider use of a flow diagram	NA
Descriptive data	14*	(a) Give characteristics of study participants (eg demographic, clinical, social) and information on exposures and potential confounders	pp.8 paragraph 2
		(b) Indicate number of participants with missing data for each variable of interest	pp.8 paragraph 2
Outcome data	15*	Report numbers of outcome events or summary measures	pp.8

For peer review only

For peer review only - http://bmjopen.bmj.com/site/about/guidelines.xhtml

			paragraph 3
Main results	16	(*a*) Give unadjusted estimates and, if applicable, confounder-adjusted estimates and their precision (eg, 95% confidence interval). Make clear which confounders were adjusted for and why they were included	pp.8 paragraph 4
		(*b*) Report category boundaries when continuous variables were categorized	NA
		(*c*) If relevant, consider translating estimates of relative risk into absolute risk for a meaningful time period	pp.8 table 3
Other analyses	17	Report other analyses done—eg analyses of subgroups and interactions, and sensitivity analyses	Table 2
**Discussion**			
Key results	18	Summarise key results with reference to study objectives	pp.9 paragraph 2
Limitations	19	Discuss limitations of the study, taking into account sources of potential bias or imprecision. Discuss both direction and magnitude of any potential bias	pp.11 paragraph 3
Interpretation	20	Give a cautious overall interpretation of results considering objectives, limitations, multiplicity of analyses, results from similar studies, and other relevant evidence	pp.10 paragraph 4-5
Generalisability	21	Discuss the generalisability (external validity) of the study results	pp.11 paragraph 5
**Other information**			
Funding	22	Give the source of funding and the role of the funders for the present study and, if applicable, for the original study on which the present article is based	pp.12

*Give information separately for exposed and unexposed groups.

Note: An Explanation and Elaboration article discusses each checklist item and gives methodological background and published examples of transparent reporting. The STROBE checklist is best used in conjunction with this article (freely available on the Web sites of PLoS Medicine at http://www.plosmedicine.org/, Annals of Internal Medicine at http://www.annals.org/, and Epidemiology at http://www.epidem.com/). Information on the STROBE Initiative is available at www.strobe-statement.org.

For peer review only

For peer review only - http://bmjopen.bmj.com/site/about/guidelines.xhtml

BMJ Open

Trends in the prevalence and treatment of depressive symptoms in Peru: A population-based study

Journal:	*BMJ Open*
Manuscript ID	bmjopen-2020-036777.R2
Article Type:	Original research
Date Submitted by the Author:	11-May-2020
Complete List of Authors:	Villarreal-Zegarra, David; Instituto Peruano de Orientación Psicológica; Universidad Peruana Cayetano Heredia, CRONICAS Center of Excellence in Chronic Diseases<br>Cabrera-Alva, Milagros; Instituto Peruano de Orientación Psicológica<br>Carrillo-Larco, Rodrigo; Universidad Peruana Cayetano Heredia, CRONICAS Center of Excellence in Chronic Diseases; Imperial College London School of Public Health, Department of Epidemiology and Biostatistics<br>Bernabe-Ortiz, Antonio; Universidad Peruana Cayetano Heredia, CRONICAS Centre of Excellence in Chronic Diseases; Universidad Científica del Sur
**Primary Subject Heading**:	Mental health
Secondary Subject Heading:	Public health, Epidemiology
Keywords:	Depression & mood disorders < PSYCHIATRY, EPIDEMIOLOGY, PUBLIC HEALTH

SCHOLARONE™
Manuscripts

I, the Submitting Author has the right to grant and does grant on behalf of all authors of the Work (as defined in the below author licence), an exclusive licence and/or a non-exclusive licence for contributions from authors who are: i) UK Crown employees; ii) where BMJ has agreed a CC-BY licence shall apply, and/or iii) in accordance with the terms applicable for US Federal Government officers or employees acting as part of their official duties; on a worldwide, perpetual, irrevocable, royalty-free basis to BMJ Publishing Group Ltd ("BMJ") its licensees and where the relevant Journal is co-owned by BMJ to the co-owners of the Journal, to publish the Work in this journal and any other BMJ products and to exploit all rights, as set out in our licence.

The Submitting Author accepts and understands that any supply made under these terms is made by BMJ to the Submitting Author unless you are acting as an employee on behalf of your employer or a postgraduate student of an affiliated institution which is paying any applicable article publishing charge ("APC") for Open Access articles. Where the Submitting Author wishes to make the Work available on an Open Access basis (and intends to pay the relevant APC), the terms of reuse of such Open Access shall be governed by a Creative Commons licence – details of these licences and which Creative Commons licence will apply to this Work are set out in our licence referred to above.

Other than as permitted in any relevant BMJ Author's Self Archiving Policies, I confirm this Work has not been accepted for publication elsewhere, is not being considered for publication elsewhere and does not duplicate material already published. I confirm all authors consent to publication of this Work and authorise the granting of this licence.

For peer review only

Trends in the prevalence and treatment of depressive symptoms in Peru: A population-based study

Authors
David Villarreal-Zegarra [1, 2]
Milagros Cabrera-Alva [2]
Rodrigo M. Carrillo-Larco [1, 3, 4]
Antonio Bernabe-Ortiz [1, 5]

Affiliation
[1] CRONICAS Center of Excellence in Chronic Diseases, Universidad Peruana Cayetano Heredia, Lima, Peru.
[2] Instituto Peruano de Orientación Psicológica, Lima, Peru.
[3] Department of Epidemiology and Biostatistics, School of Public Health, Imperial College London, London, United Kingdom.
[4] Centro de Estudios de Población, Universidad Católica los Ángeles de Chimbote (ULADECH), Chimbote, Peru.
[5] Universidad Científica del Sur, Lima, Peru.

Corresponding author
David Villarreal-Zegarra
E-mail: davidvillarreal@ipops.pe
Avenida Armendáriz 445, Miraflores, Lima 18, Perú
Telf: (+511) 241-6978

Number of Figures: 2

Number of Tables: 3

Number of Supplements: 3

Number of words: 4921

For peer review only

ARTICLE SUMMARY

Objectives: This study aimed to estimate the trends in the prevalence and treatment of depressive symptoms using nationally representative surveys in Peru from 2014-2018.
Design: A secondary analysis was conducted using five nationally representative surveys carried out consecutively in the years between 2014 and 2018, including a total of 166,290 individuals.
Setting: The study was conducted in Peru.
Participants: Individuals, men and women, aged ≥15 years who participated in the selected surveys. Sampling was probabilistic using a two-stage approach.
Main outcome measures: Two versions of the Patient Health Questionnaire (PHQ-9) that focused on the presence of depressive symptoms were administered (one in the last two-week and other in the last-year). Scores ≥15 were used as the cut-off point in both versions of the PHQ-9 to define the presence of depressive symptoms. Also, the treatment rate was based on the proportion of individuals who had experienced depressive symptoms in the last-year and who had self-reported having received specific treatment for these symptoms. The age-standardized prevalence was estimated.
Results: A total of 161,061 participants were included. There was no evidence of a change in age-standardized prevalence rates of depressive symptoms at the two weeks prior to the point of data collection (2.6% in 2014 to 2.3% in 2018), or in the last-year (6.3% in 2014 to 6.2% in 2018). Furthermore, no change was found in the proportion of depressive cases treated in the last-year (14.6% in 2014 to 14.4% in 2018). Rural areas and individuals with low-level of wealth had lower proportion of depressive cases treated.
Conclusions: No changes in trends of rates of depressive symptoms or in the proportion of depressive cases treated were observed. This suggests the need to reduce the treatment gap considering social determinants associated with inequality in access to adequate therapy.

Keywords: Depression, Epidemiology, Mood disorders, Treatment, Primary care.

For peer review only

STRENGTHS AND LIMITATIONS OF THIS STUDY

- ✓ This analysis included information from five nationally representative surveys of the Peruvian population.
- ✓ Our findings were limited as screening for depression in individuals did not involve psychiatric evaluations or structured interviews. As a result, only depressive symptoms were used as indicators to identify cases.
- ✓ Only five years were evaluated and perhaps more time may be required to identify a significant trend.

For peer review only

INTRODUCTION

Depression is a significant public health issue that affects approximately 322 million people worldwide [1] and has tremendous social and economic cost implications [2 3]. Based on current projections, it is estimated that depression will be the leading cause of years lost to disability (DALY´s) by 2030 [4]. Epidemiological surveillance of the trends in depressive symptoms is of high relevance to public health as changes in the prevalence and treatment can aid researchers and stakeholders make more informed decisions on how to address this challenge.

The prevalence of depressive symptoms can vary depending on several biological, sociodemographic, or lifestyle factors. For example, the prevalence of depressive symptoms is reportedly higher in women [5], older adults [6], and people of a low socioeconomic status [7]. On the other hand, the fact that a person with depressive symptoms can access treatment also depends on the accessibility to health services. A systematic review identified that lack of human resources, centralization of the health system, and integration in primary care, are barriers to receiving appropriate treatment of depressive symptoms [8].

Currently, there is no consensus on the trends of the prevalence of depressive symptoms. A meta-analysis of 116 epidemiological investigations during the years 1990 and 2010 did not reveal changes in the prevalence of major depressive disorder [9]. Nevertheless, when assessing trends in previous years [10 11], the Global Burden of Disease Studies found a decrease of 4.9% in the age-standardized rate of depressive disorders from 2006 to 2016. In contrast, other longitudinal studies showed an increase in the prevalence of major depressive disorder [12 13]. These mixed results can be attributed to inter-country variations of several demographic and socioeconomic factors, as well as the different criteria used to define depression in research studies. Due to the lack of global consensus, country-specific estimates and trends are significant and can inform local policies and guidelines.

In Peru, the prevalence of depressive symptoms ranges from 14% in urban areas to 12.5% in rural areas [14]. Whereas the prevalence of major depressive disorders reported in the last year was 2.7%, of which a third receive minimally adequate treatment [15]. Moreover, access to treatment for those suffering from depressive disorders is very limited in low and middle-income countries, with only 1 in every 27 patients with major depressive disorder managing to receive treatment [15]. Since 2015, Peru has been implementing mental health reforms that focus on primary care and the redistribution of its resources from hospital care to community care centers [16].

It is therefore fundamental to have up-to-date metrics on the prevalence and level of treatment coverage of depressive symptoms in order to determine an appropriate response to this disease. This study aims to estimate trends in prevalence and treatment of depressive symptoms using nationally representative surveys in Peru between 2014 and 2018.

METHODS

Study design

Data from the National Demographic and Health Survey of Peru (ENDES – Spanish acronym), which collects information on several variables, including poverty, fertility, violence, and health, was utilized in this study. Implementation of ENDES started in 1996 and was originally carried out every four years, with the second being conducted in 2000, and the third in 2004. Since 2013, ENDES began to incorporate questions regarding mental health into its evaluation. However, it initially only used a very small sub-sample that was not representative of the general population. From 2014 onwards, the data collected on mental health was nationally and regionally representative. Our study therefore decided to use data collected by ENDES between the years 2014 and 2018.

For peer review only

The data collection from each of the years included in the study used a face-to-face approach and started in February or March, with completion ending in December [17 18]. Data collection was face-to-face, with each participant evaluated being interviewed.

The participants from whom the data was being collected from in the annual ENDES evaluations were different each year. Therefore, the data being evaluated in this study came from five different groups (one from each year of evaluation). So, while there was a chance that the same participant would participate in two different years or that two or more participants from the same household would be evaluated in one year, this probability was minuscule.

Participants

The sampling used was probabilistic in two stages and representative at both national and regional levels. The sampling frame in the first stage was the selection of primary sampling units (clusters) based on information from the last census conducted in Peru. In the second stage, the selection of secondary sampling units (households) was carried out based on the information from cartographic updates and the register of buildings and households made previously [17].

In rural areas, the primary sampling units were comprised of groups ranging from 500 to 2,000 people, whereas the secondary units included households. Contrastingly, the primary sampling units in urban areas were blocks or groups of blocks involving more than 2,000 individuals with an average of 140 households. Secondary sampling units were the same as in rural settings [17]. Details on the sampling process can be found in the technical documents of the ENDES [18].

Information on the dates of birth of all household members and the order in which these data were collected was used for the selection of participants. Only one participant aged 15 years or older was selected from each household. The participant with the closest birthday to the evaluation date was selected in the ENDES. In the event of a tie between two or more participants' birthdays (i.e. same birth day), the participant whose data was collected first was selected [19].

Participants of both sexes, who were of more than 15 years of age, living in both rural and urban areas and from every region in Peru, were included in ENDES. This study only included participants with complete data on the PHQ-9 and the sociodemographic variables of interest (sex, age, area, economic level, region, and year of evaluation).

The number of participants evaluated by the ENDES in each year is about 30,000 (see Table 1).

Variables

Depressive symptomatology in the last two weeks

Depressive symptomatology was evaluated using the Patient's Health Questionnaire (PHQ-9), available in the ENDES 2014-2018. The PHQ-9 was adapted for the population in Peru and had shown optimal values of reliability and validity when tested [20]. This tool has nine items based on the DSM-IV criteria and qualifies the response options from zero to three. PHQ-9 can, therefore, present a minimum score of 0 and a maximum score of 27. Depending on the total score, the PHQ-9 results may be: without indicators of depressive symptoms (score 0 to 4); mild depressive symptoms (score 5 to 9); moderate depressive symptoms (score 10 to 14); moderate-severe depressive symptoms (score 15 to 19); and severe depressive symptoms (score 20 and above) [21].

For this study, scores of ≥15 were used to define the presence of depressive symptoms, as this cut-off point allows for better values of sensitivity (68%) and specificity (95%) [21]. In Peru, no studies have been conducted on the sensitivity and specificity of PHQ-9. Scores ≥15 were used to define the presence of depressive symptoms since it is an indicator of the presence of a major depressive episode that requires treatment [21 22].

For peer review only - http://bmjopen.bmj.com/site/about/guidelines.xhtml

Depressive symptomatology in the last year

A modified version of the PHQ-9 was used to assess depressive symptoms over the last year to help identify the occurrence of depressive symptoms over a more extended period of time. This version of the PHQ-9 was used in ENDES 2014-2018 to evaluate depressive symptoms experienced at some point in the last 12 months. Participants were asked to remember an event in the last 12 months in which they had discomfort or problems. Once the participants identified that troublesome or annoying event, the PHQ-9 was applied based on the two weeks around that event. The definition of depressive symptoms experienced some time in the last year was based on two criteria: 1) a score of ≥15 in the two-week version of PHQ-9, or 2) a score of ≥15 in the modified version of PHQ-9 for the last year. The age-standardized prevalence was also analyzed.

Proportion of depressive cases treated in the last year

A participant was considered to receive treatment for symptoms of depression based on two criteria:

1) Depressive symptoms had been recorded in the previous year or the past two weeks (PHQ-9 ≥ 15)
2) They had self-reported receiving treatment for depression from a health professional in the last 12 months.

There were three response options (“yes”, “no”, or “I do not remember”). Participants were only considered having received treatment if an affirmative answer was provided. It should be noted that ENDES data did not include information about the frequency or type of treatment received.

Other variables

In addition to the previous variables mentioned, a set of sociodemographic variables were also taken into consideration. The wealth level of participants was defined in quintiles (very low, low, middle, high, and very high) based on a wealth index available in the ENDES [23]. This index was calculated by using the availability of goods and services, the housing characteristics that the participants reported having [17]. The index was built for each year and categorized into quintiles. The calculation of this index can be found in Rutstein and Johnson [23]. As a continuous variable, age was split into four groups (15 to 34, 35 to 54, 55 to 74, and 75+). The sex (male vs. female), the study area (urban/rural), and the year of ENDES evaluation were also taken into consideration.

Statistical methods

Main analysis

Firstly, the number and proportion of participants excluded from analyses were recorded (see Supplement 1). Secondly, a descriptive analysis of the participants was carried out for each year of the ENDES. Thirdly, the age-standardized prevalence of depressive symptoms was estimated using the World Health Organization (WHO) population as the reference population [24]. The age-standardized prevalence was estimated since it enabled us to compare our results with studies conducted in other countries. A 95% confidence interval (95% CI) was calculated for the prevalence at both regional and national levels. Then, an analysis was conducted on subgroup participants reporting depressive symptoms over the past year in order to determine the proportion of those who reported receiving treatment (i.e. sex, area, age groups). Finally, the trend over time was age-standardized and then evaluated using the score test for trend; for this, the year 2014 was used as the reference category. The trend test compares the odds of cases in one year with the odds of cases in the next year. This test assumes that the trend is linear and can be used in STATA with the "tabodds" command [25].

For peer review only

Sub-analysis

Four sub-analyses were conducted to complement the main results. In order to evaluate the measurement properties of the modified version of the PHQ-9 that collects information on depressive symptoms in the last year, a sub-analysis was conducted (see Supplement 2). A confirmatory factor analysis was performed to evaluate the validity of the modified version of the PHQ-9, considering the ordinal nature of the items and using the estimator of weighted least squares means and variance adjusted [26]. These analyses evaluate whether the instrument fits the one-dimensional model proposed by the PHQ-9 as the one-dimensional model seems to be adequate[20], when optimal values are reached in the different goodness-of-fit indices. The Comparative Fit Index (CFI) and the Tucker-Lewis Index (TLI) were used; these indices must be greater than 0.90 in order to be considered of an adequate level [27]. The Root Mean Square Error of Approximation (RMSEA) with a confidence interval of 90% and the Standardized Root Mean Square Residual (SRMR) were also used, both indices considered fair values as those lower than 0.08 [27]. On the other hand, reliability was evaluated by the internal consistency coefficient of alpha and omega. Both coefficients consider that adequate levels of reliability are reached if they score higher than 0.80 [28].

An additional post hoc analysis was performed, which requires participants with depressive symptoms to meet the DSM-5 criteria. This analysis aimed to identify whether using the clinical criteria proposed by DSM-5 can alter the results (higher or lower) or be equivalent to our main results, which use a score of ≥15 in the PHQ-9. For this sub-analysis participants must have feelings of sadness or anhedonia (have a score of two or more on items 1 and 2 of the PHQ-9, i.e., “more than half the days” and “nearly every day”) and at least five of the other seven indicators (have a score of at least one on five of the other seven items of the PHQ-9).

In addition, a sub-analysis was performed to compare sociodemographic characteristics in people receiving treatment for depressive symptoms in the last year. The Pearson χ^2 test was used to make these comparisons. The generalized linear model assumed a Poisson distribution (crude and adjusted models). Assuming a Poisson distribution, link log, and robust variance were used as suggested in the literature [29]. Prevalence ratios (PR) and 95% CI were reported.

Finally, the age-standardized prevalence of depressive symptoms in the last two weeks and in the last year, and the proportion of depressive cases treated for each region of Peru were evaluated (Supplement 3).

Software used

All statistical analyses were performed using Stata 13 (StataCorp, College Station, TX, USA). The graphics were elaborated using the ggplot libraries in R (version 3. 5. 1) and QGIS v2.18. All the analyses performed considered the design by a complex sampling of the ENDES, and the analyses performed were adjusted based on the weight factor provided by each ENDES assessment year. Adjustments were made with the STATA command "svy" for all analyses, except for the factor analysis (sub-analysis) where the “lavaan” and “lavaan.survey” package was used in R.”

Ethics

The data used in our study are openly accessible to the general public. They do not use any personal identifiers (anonymous) and consequently do not represent an ethical risk for participants. The National Institute of Statistics and Informatics, a Peruvian government organization, was responsible for the collection of ENDES data. This institution requested the consent of participants to obtain the information required in the survey. The informed consent for the collection of information was taken from each person of legal age (18 years old and above). In

For peer review only

the case of minors (17 years old and younger), the request for consent was read to one of their parents or legal guardians to allow the evaluation of the minor.

Patient and Public Involvement

The patients or members of the public were not involved in the design, conduct, reporting, or dissemination plans of our research.

RESULTS

Participants

The ENDES reports published between 2014 and 2018 included the data of 166,290 participants. The participation rate varied between 95.7% in 2014 and 97.4% in 2018 (average = 96.8%). After excluding all records with incomplete information (n = 5,229, see Supplement 1), a total of 161,061 participants were included. Of these, 51.7% were women, the mean age was 40.3 years (SD = 17.1), and 73% lived in urban settings. The sociodemographic characteristics of the participants for each year are shown in Table 1.

The measurement properties of the modified version of the PHQ-9 used to assess depressive symptoms during the last year were evaluated before the primary analyses. The modified version of the PHQ-9 was identified as having evidence of validity by confirmatory factor analysis (CFI>0.90; RMSEA<0.05) and evidence of reliability by internal consistency (ω and α >0.85). More information on the factorial analysis of the modified version of the PHQ-9 for year is presented in supplement 2. These results support that the modified version of the PHQ-9 presents evidence of structural validity, supporting the one-dimensional model.

Prevalence of depressive symptomatology

In Peru, between 2014 and 2018, the age-standardized prevalence of depressive symptoms in the last two weeks (score test for trend: p-value = 0.39, χ^2=0.72) and in the last year (score test for trend: p-value = 0.38, χ^2=0.74) did not show a significant trend. In 2018, the age-standardized prevalence in the last two weeks and in the last year were 2.3% (n=892, 95% CI: 2.1%−2.5%) and 6.2% (n=2,291, 95% CI: 5.8%−6.6%), respectively (See Figure 1A). On the other hand, in 2018, the un-standardized prevalence of depressive symptoms in the last two weeks was 2.7% (95% CI: 2.4%-2.9%) and in the last year was 6.2% (95% CI: 5.8%−6.6%) and therefore showed little to no difference from the age-standardized prevalence results.

Women had a higher age-standardized prevalence of depressive symptoms in the last two weeks and the last year in comparison to men. Adults over the age of 75 years and individuals in the lowest wealth quintile were those who exhibited the highest age-standardized prevalence in the last two weeks and the last year (see Table 2). Figure 2A and Figure 2B show the age-standardized prevalence of depressive symptoms in the last two-week and last year by region, respectively. There was no variation in the trends over time in any of the evaluated regions.

Trend results were not different when DSM-5 criteria were used. Thus, the age-standardized prevalence was very similar for both depressive symptoms in the last two weeks (3.4% in 2014 to 3.3% in 2018) and in the last year (6.8% in 2014 to 6.8% in 2018).

Proportion of depressive cases treated

At the national level, the proportion of depressive cases treated in the last year did not show any significant changes over time (score test for trend: p-value = 0.19, χ^2=1.66; see figure 1B). In 2018, 14.4% (n= 292, 95% CI: 12.2%−16.9%) of people with depressive symptoms in the last year reported having received some type of treatment from a healthcare professional. A higher proportion of women self-reporting for treatment was found when compared to men (only 2018,

For peer review only - http://bmjopen.bmj.com/site/about/guidelines.xhtml

15.9% in women and 11.1% in men). However, this was not found to be statistically significant (see Table 3).

The individuals in the highest wealth quintile (26.1% in 2018) have a much higher proportion of depressive cases treated than those in the lowest wealth quintile (6.2% in 2018). The probability of receiving treatment was almost five times greater in the highest wealth quintile compared to the lowest wealth quintile (RP=5.82, 95% IC: 2.54−10.18, see Table 3). No trend of sociodemographic characteristics between 2014 and 2018 was found. At the regional level, the highest proportion of depressive cases treated was on the coast (see figure 2C). The regions with the highest proportion of depressive cases treated were Callao (38.5%), and La Libertad (28.8%). Those with the lowest proportion of depressive cases treated were Puno (4.8%) and Huancavelica (5.5%), which are regions both in located the highlands (see Supplement 3).

When performing the subanalysis that includes the DSM-5 criteria, the trend results are not modified, and the probability of receiving treatment was very similar between each other (14.5% in 2014 to 13.0% in 2018).

DISCUSSION

Main results

In Peru, from 2014 to 2018, no changes in prevalence rates (in the two weeks prior and over the last year) were found. Similarly, no change in the trends of treatment rates was found. The proportion of people with depressive symptoms receiving treatment was lower in people who live in rural areas and who are of a low level of wealth. Therefore, this situation could be generating a case of inequality in access to treatment in Peru, related to social determinants such as wealth and geographical location.

The results from this study, therefore, provide evidence for a need for an increased commitment and focus on the mental health reforms recently initiated by the Peruvian Ministry of Health. Despite strong evidence of a high prevalence of depression in the country, treatment rates remain low. Along with significant socioeconomic inequalities across the country, this requires an increased allocation of funds and additional resources to prevent and treat depression in the population.

Comparison with other studies

Prevalence of depressive symptomatology

In comparison to other countries in Latin America, Peru has demonstrated a lower (un-standardized) prevalence of depressive symptoms than Brazil (9.7%) while sharing similar levels to those found in Colombia (5.1%) [30 31]. These findings have additionally been supported by other studies [1]. Contrastingly, high-income countries such as Australia (9.8%) and the United States (8.4%) have exhibited higher age-standardized prevalences than those found in Peru [32 33]. Other countries have reported that high-income countries, such as the USA and Australia, have a population with a higher proportion of people suffering from non-communicable and chronic diseases. There is evidence that people with chronic and non-communicable diseases have a higher proportion of depressive symptoms [34], hence the higher prevalence estimate in high-income countries. It should be noted that these studies may be overestimating the proportion of depressive symptoms, as the self-report instruments tend to have higher values than structured diagnostic interviews [35].

No variation in the prevalence of depressive symptoms was identified in Peru between 2014 and 2018. Our results are consistent with a meta-analysis of 116 epidemiological studies (1990 to 2010) [9] and with other epidemiological studies conducted in Chile (2003 vs. 2010) and Germany (1997–1999 vs. 2009–2012) where only two measurements were used over a period of several

For peer review only

years [36 37]. Contrastingly, three population-based studies found that there has been an increase in depressive symptoms. The first was carried out in the USA and measured prevalence over a period of 11 years (2005 to 2015), the second was conducted for two years in Denmark (2000 vs. 2006), and the last took place in northeast Germany between 1997-2001 and 2008-2012 [12 13 38]. The heterogeneity presented in these results can be explained by the variation in the number of measurements and the amount of time evaluated between the different studies. In order to obtain a stable trend, it is necessary to have consecutive measurements. Although our work only analyzed between 2014 and 2018, it is perhaps the first to report estimates across consecutive years using nationally representative surveys.

It should be noted that the studies, as mentioned earlier, evaluate only depressive symptoms and not major depressive disorders.

Proportion of depressive cases treated

The treatment gap reported by the Peruvian Ministry of Health (85.9% in 2017) is consistent with our results [39]. However, the treatment gap is higher than those reported in other countries such as the USA (72.4%) [40] or Chile (78.8%) [36]. This larger gap could be attributed to the stigma associated with seeking mental health care and treatment, preventing patients actually to seek professional attention [41]. Limited resources and a lack of trained health personnel could also play a role [39 42].

No significant variation was found by year in the proportion of depressive cases treated in Peru. This situation is different from that of USA [43] and the Republic of Georgia [44], where there has been a significant increase in the proportion of depressive cases treated. The difference could be attributed to the new mental health policies that these countries have implemented and the amount of money they invest in mental healthcare. Peru is undergoing a mental health reform focused on primary care with the implementation of Community Mental Health Centers (CMHCs)[16]. The effects at the population level, especially with regards to the proportion of the population with depressive symptoms receiving treatment has not yet been recorded. This could be because it is too early to observe the effects of the CMHCs, as this policy started in 2015 with 22 CMHCs, whereas by the end of 2018, there were 106 CMHCs throughout Peru. Besides, CMHCs are not distributed in a decentralized manner. In the region of Lima, there are only three specialized psychiatric hospitals in Peru and 20% of all CMHCs, and this could explain why the Callao region (with only 147 km² of surface and located very close to Lima) has the highest proportion of depressive cases treated (23.8%). This region, despite its small size, has 3 CMHCs. Moreover, it is in close proximity to the three psychiatric hospitals in Peru and also other CMHCs. In comparison with Puno, which has the lowest proportion of depressive cases treated (4.8%), which is possibly due to the fact that it only has 2 CMHCs to supply a territory of 66,997 km². Although largely speculative, these data suggest that the treatment gap could be reduced if CMHCs and other mental health services were widely available in other regions.

Prevalence and treatment of depressive symptomatology

It could be argued that increasing the proportion of people receiving treatment can help reduce the prevalence of depressive symptoms, but this does not seem to be supported by the evidence. A study conducted in four high-income countries identified that a higher proportion of care is not related to reducing the prevalence of mental health problems [45]. On the other hand, a review suggests that the prevalence of depressive symptoms is reduced if the quality of mental health care is improved, waitlists are eradicated, antidepressants are prescribed with greater caution, or psychological treatments are opted for, and preventive interventions linked to the community are carried out [46].

For peer review only

Relevance in public health

The prevalence of depressive symptoms in the last year in Peru was similar to other countries; however, only 14.1% of first-level care centers provide mental health services [39]. This situation represents a public health concern as the lack of access to treatment is considered one of the causes of the treatment gap of mental health experienced by the Peruvian health system [42]. The Peruvian mental health reform seeks, among other objectives, to improve access to mental health care. Nonetheless, our results indicate that it is still too early to see the effects of this reform as the treatment gap has not been reduced at the population level in recent years (between 2014 to 2018), at least not in people with depressive symptoms. Despite this, the implementation of CMHCs seems to be a beneficial policy since the health networks that implemented a CMHC increased up to four times the number of mental health care consultations, compared to before its implementation [39]. Based on this, we could expect that the implementation of new CMHCs might increase the number of those suffering from mental health problems being attended to, and thereby reduce the treatment gap.

There is evidence that people with a clinical diagnosis of depression are more likely to receive treatment [47], and the implementation of screening for mental health problems in primary care, this has proven to be very useful in other countries to improve the diagnosis and follow-up [48 49]. However, population-based screening for depressive symptoms –or other mental health problems– has not been included in any Peruvian clinical guidelines [50 51]. Even though CMHCs aim to screen for mental health problems, their capacity is still limited and therefore it is not feasible to cover large populations. Even if other primary care facilities conducted this screening initiative, there is a limited number of psychologists, psychiatrists, and primary care physicians who can perform these screening tests [42]. It is therefore necessary for the design and implementation of a population-based screening which will require the involvement of lay people (e.g., community health workers) to fill the gap in trained health personnel. Finally, in order for a clinical practice guideline to be successfully implemented and accepted by the patients, they should be involved in the development of these guidelines [52].

Strengths and limitations

The present study includes the analysis of five measurements from different years of information collected from nationally-representative surveys in the Peruvian population . This is the first study assessing the trends of depressive symptoms in Peru. However, this study has limitations. Firstly, the evaluation period may have been too short (between 2014 to 2018). If prevalence or treatment were to increase, it most likely is small but sustained annually. It would therefore be to have a higher number of measurements (10 or 20 years) in order to be able to identify a trend of higher or a decrease of depression symptoms. Secondly, although the evaluation of depressive symptoms was performed using a valid tool [53], this does not replace an evaluation conducted by a psychiatrist; thus, misclassification may be an issue. Thirdly, another element that could generate bias is the cut-off point used to classify people with depressive symptoms (PHQ-9 scores ≥15). However, when the post hoc analysis was performed using the DSM-5 criteria, the main results were identified as the same (no change in prevalence trends and no change in the proportion of depressive cases treated). Fourth, possible loss mechanisms in the missing data were not evaluated. Fifth, the modified version of the PHQ-9 (in the last year) may have introduced measurement bias. Although evidence of reliability and validity was presented in this study by internal structure, further studies are required to obtain other evidence of validity (i.e. relationship to other variables, invariance, sensitivity/specificity). In particular, studies on the measurement validity of the modified version of the PHQ-9 are required. Finally, other important variables that would have allowed a better understanding of the results were not included. For

For peer review only

example, type of treatment received, duration of treatment, and if the participant has previously received a diagnosis of depression.

Conclusions

No significant trend was found in the age-standardized prevalence of depressive symptoms (in the last two weeks and the last year) or in the proportion of depressive cases treated. The proportion of depressive cases treated was lower in people with depressive symptoms who lived in rural areas and who were of a low level of wealth. It is therefore necessary to implement policies and interventions in order to reduce the treatment gap and the prevalence of depressive symptoms that can be attributed to social inequalities across the country.

CONTRIBUTORSHIP STATEMENT

David Villarreal-Zegarra: Conceived and designed the analysis; collected the data; contributed data or analysis tools; performed the analysis; wrote the paper.

Milagros Cabrera-Alva: Contributed data or analysis tools; wrote the paper.

Rodrigo M Carrillo-Larco: Conceived and designed the analysis; contributed data or analysis tools; performed the analysis; wrote the paper; supervision of the paper.

Antonio Bernabe-Ortiz: Conceived and designed the analysis; contributed data or analysis tools; performed the analysis; wrote the paper; supervision of the paper.

COMPETING INTERESTS

The authors have declared that no competing interests exist.

FUNDING

This work was supported by the Strategic Award, Wellcome Trust-Imperial College Centre for Global Health Research (100693/Z/12/Z), Imperial College London Wellcome Trust Institutional Strategic Support Fund [Global Health Clinical Research Training Fellowship] (294834/Z/16/Z ISSF ICL), and Rodrigo M Carrillo-Larco are supported by a Wellcome Trust International Training Fellowship (214185/Z/18/Z). The funders had no role in the study design, data collection, analysis, decision to publish, or preparation of the manuscript.

GRANT DISCLOSURES

The authors disclosed the following grant information:

Strategic Award, Wellcome Trust-Imperial College Centre for Global Health Research: 100693/Z/12/Z.

Imperial College London Wellcome Trust Institutional Strategic Support Fund: 294834/Z/16/Z ISSF ICL.

Wellcome Trust International Training Fellowship: 214185/Z/18/Z.

DATA SHARING STATEMENT

The database is freely accessible from the website of the National Institute of Statistics of Peru, URL: http://iinei.inei.gob.pe/microdatos/

The information can be obtained by entering the survey query tab and selecting the ENDES data using the health module data. Only cross-sectional information from 2014 to 2018 for the ENDES Health Questionnaire was used.

For peer review only

ACKNOWLEDGMENTS
DVZ thanks Helena Kesar and Qory Moscoso for their style review in the final version of the English manuscript.

ABBREVIATIONS

CFI: Comparative Fit Index.
CMHCs: Community Mental Health Centers.
DSM-IV: Diagnostic and Statistical Manual of Mental Disorders, 4th Edition.
DSM-5: Diagnostic and Statistical Manual of Mental Disorders, 5th Edition.
ENDES: Demographic and Health Survey of Peru.
PHQ-9: Patient's Health Questionnaire.
PR: Prevalence Ratios.
RMSEA: Root Mean Square Error of Approximation.
SD: Standard Deviation.
SRMR: Standardized Root Mean Square Residual.
TLI: Tucker-Lewis Index.
USA: United States of America.
WHO: World Health Organization.
95%CI: 95% confidence interval.

REFERENCES

1. World Health Organization. Depression and Other Common Mental Disorders. Global Health Estimates. Geneve: World Health Organization 2017.
2. Hsieh CR, Qin X. Depression hurts, depression costs: The medical spending attributable to depression and depressive symptoms in China. *Health economics* 2018;27(3):525-44. doi: 10.1002/hec.3604 [published Online First: 2017/10/11]
3. Vasiliadis HM, Dezetter A, Latimer E, et al. Assessing the Costs and Benefits of Insuring Psychological Services as Part of Medicare for Depression in Canada. *Psychiatric services* 2017;68(9):899-906. doi: 10.1176/appi.ps.201600395 [published Online First: 2017/05/16]
4. World Health Organization. The global burden of disease: 2004 update. Geneva: WHO 2008.
5. Salk RH, Hyde JS, Abramson LY. Gender differences in depression in representative national samples: Meta-analyses of diagnoses and symptoms. *Psychol Bull* 2017;143(8):783-822. doi: 10.1037/bul0000102 [published Online First: 2017/04/28]
6. Shao J, Li D, Zhang D, et al. Birth cohort changes in the depressive symptoms of Chinese older adults: a cross-temporal meta-analysis. *International Journal of Geriatric Psychiatry* 2013;28(11):1101-08. doi: doi:10.1002/gps.3942
7. Lee E-W, Park J-H. Individual and Socioeconomic Contextual Effects on Depressive Symptom in Korea: Multilevel Analysis of Cross-sectional Nationwide Survey. *Journal of Korean medical science* 2015;30(2):186-93.
8. Eaton J, McCay L, Semrau M, et al. Scale up of services for mental health in low-income and middle-income countries. *Lancet (London, England)* 2011;378(9802):1592-603. doi: 10.1016/s0140-6736(11)60891-x [published Online First: 2011/10/20]
9. Baxter AJ, Scott KM, Ferrari AJ, et al. Challenging the myth of an "epidemic" of common mental disorders: trends in the global prevalence of anxiety and depression between 1990 and 2010. *Depression and Anxiety* 2014;31(6):506-16. doi: doi:10.1002/da.22230
10. GBD 2016 Disease and Injury Incidence and Prevalence Collaborators. Global, regional, and national incidence, prevalence, and years lived with disability for 328 diseases and injuries

For peer review only

for 195 countries, 1990-2016: a systematic analysis for the Global Burden of Disease Study 2016. *Lancet (London, England)* 2017;390(10100):1211-59. doi: 10.1016/s0140-6736(17)32154-2 [published Online First: 2017/09/19]

11. GBD 2015 Disease and Injury Incidence and Prevalence Collaborators. Global, regional, and national incidence, prevalence, and years lived with disability for 310 diseases and injuries, 1990-2015: a systematic analysis for the Global Burden of Disease Study 2015. *The Lancet* 2016;388(10053):1545-602. doi: 10.1016/s0140-6736(16)31678-6 [published Online First: 2016/10/14]
12. Andersen I, Thielen K, Bech P, et al. Increasing prevalence of depression from 2000 to 2006. *Scandinavian Journal of Public Health* 2011;39(8):857-63. doi: 10.1177/1403494811424611
13. Weinberger AH, Gbedemah M, Martinez AM, et al. Trends in depression prevalence in the USA from 2005 to 2015: widening disparities in vulnerable groups. *Psychological medicine* 2018;48(8):1308-15. doi: 10.1017/s0033291717002781 [published Online First: 2017/10/13]
14. Guerra M, Prina AM, Ferri CP, et al. A comparative cross-cultural study of the prevalence of late life depression in low and middle income countries. *Journal of Affective Disorders* 2016;190:362-68. doi: https://doi.org/10.1016/j.jad.2015.09.004
15. Thornicroft G, Chatterji S, Evans-Lacko S, et al. Undertreatment of people with major depressive disorder in 21 countries. *British Journal of Psychiatry* 2018;210(2):119-24. doi: 10.1192/bjp.bp.116.188078 [published Online First: 01/02]
16. Toyama M, Castillo H, Galea JT, et al. Peruvian Mental Health Reform: A Framework for Scaling-up Mental Health Services. *International journal of health policy and management* 2017;6(9):501-08. doi: 10.15171/ijhpm.2017.07 [published Online First: 2017/09/28]
17. Instituto Nacional de Estadística e Informática. Perú: encuesta demográfica y de salud familiar ENDES 2014. Perú: Instituto Nacional de Estadística e Informática 2015.
18. Instituto Nacional de Estadística e Informática. Perú - Encuesta Demográfica y de Salud Familiar ENDES 2017. Peru: Instituto Nacional de Estadística e Informática 2018.
19. Instituto Nacional de Estadística e Informática. Manual de la entrevistadora - Encuesta demográfica y de salud familiar. Perú: Instituto Nacional de Estadística e Informática 2018.
20. Villarreal-Zegarra D, Copez-Lonzoy A, Bernabe-Ortiz A, et al. Valid group comparisons can be made with the Patient Health Questionnaire (PHQ-9): A measurement invariance study across groups by demographic characteristics. *PloS one* 2019;14(9):e0221717. doi: 10.1371/journal.pone.0221717 [published Online First: 2019/09/10]
21. Kroenke K, Spitzer RL, Williams JB. The PHQ-9: validity of a brief depression severity measure. *J Gen Intern Med* 2001;16(9):606-13. [published Online First: 2001/09/15]
22. Manea L, Gilbody S, McMillan D. A diagnostic meta-analysis of the Patient Health Questionnaire-9 (PHQ-9) algorithm scoring method as a screen for depression. *General hospital psychiatry* 2015;37(1):67-75. doi: 10.1016/j.genhosppsych.2014.09.009 [published Online First: 2014/12/03]
23. Rutstein SO, Johnson K. The DHS wealth index. DHS comparative reports no. 6. Calverton, Maryland: ORC Macro 2004.
24. Ahmad OB, Boschi-Pinto C, Lopez AD, et al. Age standardization of rates: a new who standard. World Health Organization: Geneva 2001.
25. Clayton DH, Michael. Statistical Models in Epidemiology. Oxford: Oxford University Press 1993.
26. Brown TA. Confirmatory factor analysis for applied research. Second ed. New York: The Guilford Press 2015.
27. Hair JFA, R.E.; Tatham, R.L. & Black, W. Análisis Multivariante. Madrid: Prentice Hall 1999.

For peer review only

28. Ten Berge JM, Sočan G. The greatest lower bound to the reliability of a test and the hypothesis of unidimensionality. *Psychometrika* 2004;69(4):613-25.
29. Barros AJ, Hirakata VN. Alternatives for logistic regression in cross-sectional studies: an empirical comparison of models that directly estimate the prevalence ratio. *BMC Med Res Methodol* 2003;3(1):21.
30. Gómez-Restrepo C, Tamayo Martínez N, Bohórquez A, et al. Trastornos depresivos y de ansiedad y factores asociados en la población adulta colombiana, Encuesta Nacional de Salud Mental 2015. *Revista Colombiana de Psiquiatría* 2016;45:58-67. doi: https://doi.org/10.1016/j.rcp.2016.04.009
31. Barros MBA, Lima MG, Azevedo RCS, et al. Depression and health behaviors in Brazilian adults - PNS 2013. *Rev Saude Publica* 2017;51(suppl 1):8s. doi: 10.1590/s1518-8787.2017051000084 [published Online First: 2017/06/08]
32. Zhao G, Ford ES, Dhingra S, et al. Depression and anxiety among US adults: associations with body mass index. *International journal of obesity (2005)* 2009;33(2):257-66. doi: 10.1038/ijo.2008.268 [published Online First: 2009/01/07]
33. Atlantis E, Goldney RD, Eckert KA, et al. Trends in health-related quality of life and health service use associated with comorbid diabetes and major depression in South Australia, 1998-2008. *Social psychiatry and psychiatric epidemiology* 2012;47(6):871-7. doi: 10.1007/s00127-011-0394-4 [published Online First: 2011/05/19]
34. DeJean D, Giacomini M, Vanstone M, et al. Patient experiences of depression and anxiety with chronic disease: a systematic review and qualitative meta-synthesis. *Ontario health technology assessment series* 2013;13(16):1-33. [published Online First: 2013/11/15]
35. Lowe B, Kroenke K, Herzog W, et al. Measuring depression outcome with a brief self-report instrument: sensitivity to change of the Patient Health Questionnaire (PHQ-9). *J Affect Disord* 2004;81(1):61-6. doi: 10.1016/s0165-0327(03)00198-8 [published Online First: 2004/06/09]
36. Markkula N, Zitko P, Pena S, et al. Prevalence, trends, correlates and treatment of depression in Chile in 2003 to 2010. *Social psychiatry and psychiatric epidemiology* 2017;52(4):399-409. doi: 10.1007/s00127-017-1346-4 [published Online First: 2017/02/16]
37. Bretschneider J, Janitza S, Jacobi F, et al. Time trends in depression prevalence and health-related correlates: results from population-based surveys in Germany 1997–1999 vs. 2009–2012. *BMC psychiatry* 2018;18(1):394. doi: 10.1186/s12888-018-1973-7
38. Baumeister SE, Schomerus G, Schmidt CO, et al. Change in depressive symptoms and mental health-related quality of life in northeast Germany between 1997-2001 and 2008-2012. *International journal of public health* 2015;60(1):33-9. doi: 10.1007/s00038-014-0632-0 [published Online First: 2014/12/17]
39. Ministerio de Salud. Plan Nacional de Fortalecimiento de Servicios de Salud Mental Comunitaria 2018 – 2021. Perú: Ministerio de Salud 2018.
40. Wittayanukorn S, Qian J, Hansen RA. Prevalence of depressive symptoms and predictors of treatment among U.S. adults from 2005 to 2010. *Gen Hosp Psychiatry* 2014;36(3):330-6. doi: 10.1016/j.genhosppsych.2013.12.009 [published Online First: 2014/01/28]
41. Pattyn E, Verhaeghe M, Sercu C, et al. Public stigma and self-stigma: differential association with attitudes toward formal and informal help seeking. *Psychiatric services (Washington, DC)* 2014;65(2):232-8. doi: 10.1176/appi.ps.201200561 [published Online First: 2013/11/16]
42. Grupo del Banco Mundial. Movimiento por el cuidado comunitario de la salud mental en el Perú. Lima: Grupo del Banco Mundial 2019.

For peer review only

43. Mojtabai R, Olfson M, Han B. National Trends in the Prevalence and Treatment of Depression in Adolescents and Young Adults. *Pediatrics* 2016;138(6) doi: 10.1542/peds.2016-1878 [published Online First: 2016/12/13]
44. Kenchadze V, Chkonia E, Beria Z. Trends in the use of outpatient psychiatric services in the republic of Georgia. *Georgian medical news* 2013(215):60-5. [published Online First: 2013/03/14]
45. Jorm AF, Patten SB, Brugha TS, et al. Has increased provision of treatment reduced the prevalence of common mental disorders? Review of the evidence from four countries. *World psychiatry : official journal of the World Psychiatric Association (WPA)* 2017;16(1):90-99. doi: 10.1002/wps.20388 [published Online First: 2017/01/28]
46. Ormel J, Kessler RC, Schoevers R. Depression: more treatment but no drop in prevalence: how effective is treatment? And can we do better? *Current opinion in psychiatry* 2019;32(4):348-54. doi: 10.1097/yco.0000000000000505 [published Online First: 2019/03/12]
47. Fujii RK, Goren A, Annunziata K, et al. Prevalence, Awareness, Treatment, and Burden of Major Depressive Disorder: Estimates from the National Health and Wellness Survey in Brazil. *Value in health regional issues* 2012;1(2):235-43. doi: 10.1016/j.vhri.2012.09.011 [published Online First: 2012/12/01]
48. Siu AL, Bibbins-Domingo K, Grossman DC, et al. Screening for Depression in Adults: US Preventive Services Task Force Recommendation Statement. *Jama* 2016;315(4):380-7. doi: 10.1001/jama.2015.18392 [published Online First: 2016/01/28]
49. Vilagut G, Forero CG, Barbaglia G, et al. Screening for Depression in the General Population with the Center for Epidemiologic Studies Depression (CES-D): A Systematic Review with Meta-Analysis. *PloS one* 2016;11(5):e0155431. doi: 10.1371/journal.pone.0155431 [published Online First: 2016/05/18]
50. Ministerio de Salud. Guía de practica clínica en depresión. Perú: Ministerio de Salud 2008.
51. Instituto Nacional de Salud. Guía de práctica clínica para el tratamiento de depresión en adultos - versión extensa. Lima: Instituto Nacional de Salud 2017.
52. Zafra-Tanaka JH, Goicochea-Lugo S, Villarreal-Zegarra D, et al. Characteristics and quality of clinical practice guidelines for depression in adults: a scoping review. *BMC psychiatry* 2019;19(1):76. doi: 10.1186/s12888-019-2057-z
53. Baader M T, Molina F JL, Venezian B S, et al. Validación y utilidad de la encuesta PHQ-9 (Patient Health Questionnaire) en el diagnóstico de depresión en pacientes usuarios de atención primaria en Chile. *Rev Chil Neuropsiquiatr* 2012;50:10-22.

For peer review only

FIGURES AND TABLES

Figure 1. Age-standardized prevalence of depressive symptoms and proportion of depressive cases treated in Peru between 2014 to 2018.
A) Age-standardized prevalence of depressive symptoms in the last year and the last two weeks in Peru by year. B) Proportion of depressive cases treated in the last year in Peru by year. All the analyzes, the weighted proportion by complex sampling was used.

Figure 2. Age-standardized prevalence of depressive symptoms and proportion of depressive cases treated in Peru by region in 2018.
A) Age-standardized prevalence of depressive symptoms in the last two weeks from Peru for a region in 2018. B) Age-standardized prevalence of depressive symptoms in the last year in Peru for a region in 2018. C) Proportion of depressive cases treated in the last year for the region in 2018. Two-stage sample design was taken into account for percentage estimations.
Figure designed by the authors.

For peer review only

Table 1. Descriptive characteristics of the participants included in the study by year.

		2014 (n=27,633)		2015 (n=33,573)		2016 (n=32,373)		2017 (n=33,794)		2018 (n=34,476)	
		n	%	*n*	%	*n*	%	*n*	%	*n*	%
Sex	Male	12,806	46.3%	14,788	48.9%	14,126	48.9%	14,424	48.5%	14,696	48.4%
	Female	14,827	53.7%	18,573	51.1%	18,247	51.1%	18,794	51.5%	19,780	51.6%
Age	15-34	11,022	43.1%	16,576	43.1%	15,498	44.4%	15,913	43.6%	16,121	42.7%
	35-54	9,395	33.9%	10,887	33.9%	10,740	33.8%	10,819	34.5%	11,692	34.6%
	55-74	5,460	18.0%	4,685	18.0%	4,791	16.8%	5,030	17.0%	5,332	17.7%
	75+	1,756	5.0%	1,213	5.0%	1,344	5.0%	1,456	4.9%	1,331	5.0%
Area	Rural	10,663	24.9%	11,453	34.5%	11,113	35.2%	11,349	20.8%	11,923	19.6%
	Urban	16,970	75.1%	21,908	65.5%	21,260	64.8%	21,869	79.3%	22,553	80.4%
Wealth index	Very low	8,151	18.7%	9,370	26.3%	8,967	26.4%	10,045	18.5%	11,019	18.7%
	Low	6,782	19.2%	8,376	21.2%	8,464	22.0%	8,575	20.8%	8,514	20.6%
	Middle	5,153	19.9%	6,459	18.3%	6,361	18.5%	6,368	21.0%	6,379	20.9%
	High	4,103	21.0%	5,089	17.7%	5,056	17.4%	4,846	20.3%	4,909	20.4%
	Very high	3,444	21.2%	4,067	16.4%	3,525	15.8%	3,384	19.4%	3,655	19.4%

Note: Two-stage sample design was taken into account for percentage estimations.

For peer review only

Table 2. Age-standardized prevalence of depressive symptoms and proportion of depressive cases treated in the last year in Peru by selected socio-demographic characteristics (2014-2018, with cut-off point 15).

			2014	2015	2016	2017	2018
Age-standardized prevalence of Depressive symptoms in the last two week †	Sex	Male	1.5%	1.9%	1.9%	1.3%	1.1%
		Female	3.6%	3.9%	4.0%	3.3%	3.4%
	Age	15-34	1.5%	1.5%	1.6%	1.2%	1.1%
		35-54	2.9%	2.6%	2.6%	2.5%	2.3%
		55-74	4.0%	5.7%	5.0%	4.1%	4.1%
		75+	6.7%	9.0%	11.2%	6.0%	6.5%
	Area	Rural	2.9%	3.5%	3.4%	3.2%	3.3%
		Urban	2.5%	2.5%	2.7%	2.1%	2.0%
	Wealth index	Very low	3.0%	3.8%	3.6%	3.2%	3.5%
		Low	3.1%	3.3%	2.8%	3.1%	2.7%
		Middle	3.3%	3.2%	3.5%	2.8%	2.4%
		High	2.5%	2.1%	2.6%	1.6%	1.7%
		Very high	1.6%	1.7%	1.7%	1.2%	1.2%
Age-standardized prevalence of Depressive symptoms in the last year †	Sex	Male	3.7%	4.3%	4.0%	3.5%	3.9%
		Female	8.5%	9.1%	9.3%	8.2%	8.2%
	Age	15-34	4.5%	4.4%	4.6%	4.1%	4.3%
		35-54	6.9%	6.7%	6.5%	6.0%	6.1%
		55-74	8.1%	10.6%	9.7%	9.3%	9.0%
		75+	12.5%	15.3%	17.3%	9.6%	13.2%
	Area	Rural	7.2%	7.9%	7.6%	7.4%	8.1%
		Urban	5.9%	6.0%	6.1%	5.5%	5.6%
	Wealth index	Very low	7.4%	8.1%	7.8%	7.3%	8.4%
		Low	7.4%	7.7%	7.1%	7.4%	7.1%
		Middle	7.1%	6.9%	7.4%	6.5%	6.6%
		High	5.4%	5.7%	5.9%	4.6%	5.0%
		Very high	4.8%	4.5%	4.3%	4.3%	4.3%
Proportion of depressive cases treated in the last year ‡	Sex	Male	13.3%	11.9%	10.7%	10.6%	11.1%
		Female	15.1%	14.7%	16.9%	16.6%	15.9%
	Age	15-34	15.7%	14.8%	19.7%	19.2%	14.7%
		35-54	17.3%	16.1%	18.0%	15.9%	13.6%
		55-74	12.1%	12.2%	10.5%	10.0%	16.0%
		75+	7.4%	8.5%	6.2%	10.0%	12.5%
	Area	Rural	7.9%	7.2%	7.1%	5.6%	7.8%
		Urban	17.5%	18.6%	21.0%	18.4%	16.9%
	Wealth index	Very low	5.8%	5.7%	5.8%	3.3%	6.2%
		Low	8.8%	11.6%	14.1%	13.2%	10.1%
		Middle	16.3%	13.8%	17.1%	15.3%	17.4%
		High	20.3%	22.0%	24.7%	14.2%	20.4%
		Very high	27.1%	31.3%	31.0%	38.4%	26.1%

Note: Two-stage sample design was taken into account for percentage estimations. † The analysis considered the total of the Peruvian population. ‡ An analysis is done by subgroups, considering only people who have depressive symptoms.

For peer review only

Table 3. Association between receiving treatment and sociodemographic characteristics in people with depressive symptoms in last year, only in 2018.

		Model crude PR (95% CI)	Adjusted model * PR (95% CI)
Sex	Male	1	1
	Female	1.42 (0.96 - 2.10)	1.30 (0.88 - 1.92)
Age	15-34	1	1
	35-54	0.92 (0.64 - 1.32)	0.98 (0.69 - 1.41)
	55-74	1.09 (0.71 - 1.67)	1.23 (0.81 - 1.87)
	75+	0.85 (0.37 - 1.91)	1.08 (0.49 - 2.39)
Area	Rural	1	1
	Urban	**2.18 (1.58 - 3.00)**	0.81 (0.48 - 1.37)
Wealth index	Very low	1	1
	Low	**1.65 (1.05 - 2.60)**	**1.89 (1.06 - 3.38)**
	Middle	**2.84 (1.78 - 4.50)**	**3.36 (1.73 - 6.53)**
	High	**3.32 (2.04 - 5.40)**	**3.92 (1.96 - 7.87)**
	Very high	**4.25 (2.65 - 6.81)**	**5.08 (2.54 - 10.18)**

Note: Analysis is done by subgroups, considering only people who have depressive symptoms, the complex sampling was used.

* Adjusted by sex, age, wealth index, and area.

For peer review only

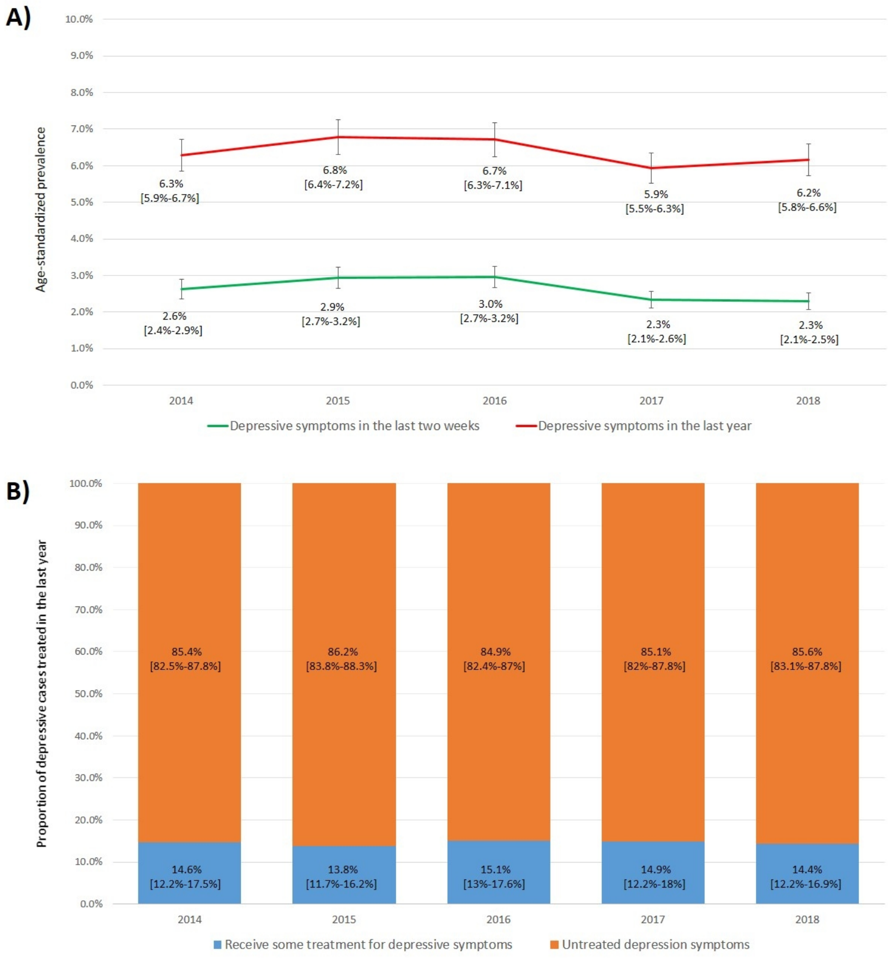

Figure 1. Age-standardized prevalence of depressive symptoms and proportion of depressive cases treated in Peru between 2014 to 2018.

350x350mm (96 x 96 DPI)

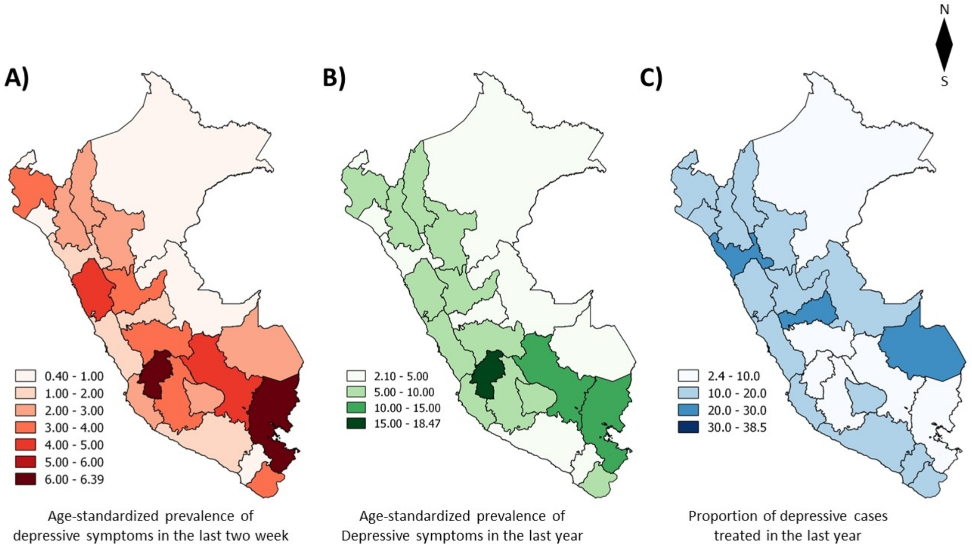

Figure 2. Age-standardized prevalence of depressive symptoms and proportion of depressive cases treated in Peru by region in 2018.

350x350mm (96 x 96 DPI)

Supplement 1. Descriptive characteristics of the participants not included in the study (2014-2018).

		2014		2015		2016		2017		2018	
		n	%	*n*	%	*n*	%	*n*	%	*n*	%
Sex	Male	0	0.0%	1	0.1%	0	0.0%	0	0.0%	0	0.00%
	Female	0	0.0%	8	0.7%	0	0.0%	1	0.1%	0	0.00%
	Missing	1,160	100.0%	1,101	99.2%	1,166	100.0%	880	99.9%	912	100.00%
Age	15-34	0	0.0%	4	0.3%	0	0.0%	0	0.0%	0	0.00%
	35-54	0	0.0%	3	0.3%	0	0.0%	0	0.0%	0	0.00%
	55-74	0	0.0%	1	0.1%	0	0.0%	0	0.0%	0	0.00%
	75+	0	0.0%	1	0.1%	0	0.0%	74	0.1%	0	0.00%
	Missing	1,160	100.0%	1,101	99.2%	1,166	100.0%	880	99.9%	912	100.00%
Area	Rural	261	22.5%	289	26.0%	253	21.7%	194	22.0%	175	19.2%
	Urban	899	77.5%	821	74.0%	913	78.3%	687	78.0%	737	80.8%
	Missing	0	0.0%	0	0.0%	0	0.0%	0	0.0%	0	0.00%
Wealth index	Very low	207	17.8%	261	23.5%	214	18.4%	181	20.5%	155	17.0%
	Low	205	17.7%	201	18.1%	209	17.9%	152	17.3%	162	17.8%
	Middle	230	19.8%	192	17.3%	232	19.9%	152	17.3%	199	21.8%
	High	241	20.8%	225	20.3%	258	22.1%	204	23.2%	159	17.4%
	Very high	277	23.9%	231	20.8%	253	21.7%	192	21.8%	237	26.0%
	Missing	0	0.0%	0	0.0%	0	0.0%	0	0.0%	0	0.00%

For peer review only

Supplement 2. Factor loads, goodness-of-fit indices, and internal consistency coefficients of the modified version of the PHQ-9 to evaluate depressive symptoms in the past year.

	2014	2015	2016	2017	2018
1. Anhedonia	0.63	0.66	0.69	0.69	0.69
2. Depressed mood	0.66	0.67	0.71	0.71	0.68
3. Sleep disturbance	0.62	0.65	0.66	0.66	0.64
4. Fatigue	0.64	0.68	0.68	0.68	0.70
5. Appetite changes	0.62	0.64	0.63	0.63	0.63
6. Feelings of worthlessness	0.66	0.67	0.68	0.68	0.71
7. Concentration difficulties	0.65	0.67	0.67	0.67	0.68
8. Psychomotor disturbances	0.55	0.59	0.60	0.60	0.58
9. Thoughts of death	0.51	0.56	0.55	0.55	0.54
χ^2	660.6	768.0	864.0	864.2	679.2
CFI	0.918	0.925	0.912	0.920	0.921
TLI	0.900	0.901	0.898	0.901	0.901
RMSEA [CI 90%]	0.051 [0.049 - 0.053]	0.053 [0.051 - 0.055]	0.058 [0.056 - 0.060]	0.059 [0.058 - 0.061]	0.050 [0.051 - 0.053]
α	0.85	0.86	0.87	0.87	0.87
ω	0.85	0.87	0.87	0.87	0.86
r with the PHQ-9 (two-week)	0.60	0.61	0.61	0.61	0.60

For peer review only

Supplement 3. Age-standardized prevalence of depressive symptoms and proportion of treatment in Peru by region in 2018 (with cut-off point 15).

Region	Age-standardized prevalence of Depressive symptoms in the last two week *	Age-standardized prevalence of Depressive symptoms in the last year *	Proportion of People with depressive symptoms receiving treatment **
Amazonas	2.19%	5.63%	11.77%
Ancash	4.03%	9.95%	10.67%
Apurímac	2.52%	7.81%	16.63%
Arequipa	1.20%	4.19%	19.51%
Ayacucho	3.68%	8.93%	8.27%
Cajamarca	2.47%	5.54%	11.48%
Callao	1.70%	4.51%	38.51%
Cusco	4.36%	12.36%	9.74%
Huancavelica	6.10%	18.47%	5.89%
Huánuco	3.62%	7.95%	17.87%
Ica	2.99%	5.78%	15.42%
Junín	3.12%	6.87%	9.79%
La Libertad	1.41%	4.15%	28.52%
Lambayeque	0.40%	2.10%	17.19%
Lima	1.69%	5.40%	15.77%
Loreto	0.80%	2.20%	2.38%
Madre de Dios	2.12%	3.63%	22.03%
Moquegua	0.84%	3.36%	10.07%
Pasco	1.27%	4.61%	24.52%
Piura	3.21%	7.91%	14.08%
Puno	6.39%	13.71%	7.12%
San Martín	2.05%	5.16%	12.92%
Tacna	3.86%	7.21%	12.34%
Tumbes	0.88%	2.52%	13.03%
Ucayali	0.75%	3.39%	14.51%

Note: Two-stage sample design was taken into account for percentage estimations. * The analysis considered the total of Peruvian population. ** An analysis is done by subgroups, considering only people who have depressive symptoms.

For peer review only

STROBE Statement—Checklist of items that should be included in reports of ***cross-sectional studies***

	Item No	Recommendation	Page No
**Title and abstract**	1	(*a*) Indicate the study's design with a commonly used term in the title or the abstract	pp.1
		(*b*) Provide in the abstract an informative and balanced summary of what was done and what was found	pp.2
**Introduction**			
Background/rationale	2	Explain the scientific background and rationale for the investigation being reported	pp.4
Objectives	3	State specific objectives, including any prespecified hypotheses	pp.4 paragraph 5
**Methods**			
Study design	4	Present key elements of study design early in the paper	pp.4
Setting	5	Describe the setting, locations, and relevant dates, including periods of recruitment, exposure, follow-up, and data collection	pp.4 paragraph 6
Participants	6	(*a*) Give the eligibility criteria, and the sources and methods of selection of participants	pp.5 paragraph 3
Variables	7	Clearly define all outcomes, exposures, predictors, potential confounders, and effect modifiers. Give diagnostic criteria, if applicable	pp.5 paragraph 8
Data sources/ measurement	8*	For each variable of interest, give sources of data and details of methods of assessment (measurement). Describe comparability of assessment methods if there is more than one group	pp.5 paragraph 8
Bias	9	Describe any efforts to address potential sources of bias	pp.5 paragraph 8
Study size	10	Explain how the study size was arrived at	NA
Quantitative variables	11	Explain how quantitative variables were handled in the analyses. If applicable, describe which groupings were chosen and why	pp.5 paragraph 8
Statistical methods	12	(*a*) Describe all statistical methods, including those used to control for confounding	pp.6
		(*b*) Describe any methods used to examine subgroups and interactions	pp.6
		(*c*) Explain how missing data were addressed	pp.7
		(*d*) If applicable, describe analytical methods taking account of sampling strategy	pp.7
		(*e*) Describe any sensitivity analyses	pp.7
**Results**			
Participants	13*	(a) Report numbers of individuals at each stage of study—eg numbers potentially eligible, examined for eligibility, confirmed eligible, included in the study, completing follow-up, and analysed	pp.8 paragraph 1
		(b) Give reasons for non-participation at each stage	pp.8 paragraph 2
		(c) Consider use of a flow diagram	NA
Descriptive data	14*	(a) Give characteristics of study participants (eg demographic, clinical, social) and information on exposures and potential confounders	pp.8 paragraph 2
		(b) Indicate number of participants with missing data for each variable of interest	pp.8 paragraph 2
Outcome data	15*	Report numbers of outcome events or summary measures	pp.8

For peer review only

			paragraph 3
Main results	16	(*a*) Give unadjusted estimates and, if applicable, confounder-adjusted estimates and their precision (eg, 95% confidence interval). Make clear which confounders were adjusted for and why they were included	pp.8 paragraph 4
		(*b*) Report category boundaries when continuous variables were categorized	NA
		(*c*) If relevant, consider translating estimates of relative risk into absolute risk for a meaningful time period	pp.8 table 3
Other analyses	17	Report other analyses done—eg analyses of subgroups and interactions, and sensitivity analyses	Table 2
**Discussion**			
Key results	18	Summarise key results with reference to study objectives	pp.9 paragraph 2
Limitations	19	Discuss limitations of the study, taking into account sources of potential bias or imprecision. Discuss both direction and magnitude of any potential bias	pp.11 paragraph 3
Interpretation	20	Give a cautious overall interpretation of results considering objectives, limitations, multiplicity of analyses, results from similar studies, and other relevant evidence	pp.10 paragraph 4-5
Generalisability	21	Discuss the generalisability (external validity) of the study results	pp.11 paragraph 5
**Other information**			
Funding	22	Give the source of funding and the role of the funders for the present study and, if applicable, for the original study on which the present article is based	pp.12

*Give information separately for exposed and unexposed groups.

Note: An Explanation and Elaboration article discusses each checklist item and gives methodological background and published examples of transparent reporting. The STROBE checklist is best used in conjunction with this article (freely available on the Web sites of PLoS Medicine at http://www.plosmedicine.org/, Annals of Internal Medicine at http://www.annals.org/, and Epidemiology at http://www.epidem.com/). Information on the STROBE Initiative is available at www.strobe-statement.org.

For peer review only

For peer review only - http://bmjopen.bmj.com/site/about/guidelines.xhtml
